# Graph reconstruction using covariance-based methods

**DOI:** 10.1186/s13637-016-0052-y

**Published:** 2016-11-23

**Authors:** Nurgazy Sulaimanov, Heinz Koeppl

**Affiliations:** 1 Department of Electrical Engineering and Information Technology, Technische Universität Darmstadt, Rundeturmstr. 12, Darmstadt, 64283 Germany; 2 Department of Biology, Technische Universität Darmstadt, Schnittspahnstr. 10, Darmstadt, 64287 Germany

**Keywords:** High-dimensional graph reconstruction methods, Concentration and covariance graphs

## Abstract

Methods based on correlation and partial correlation are today employed in the reconstruction of a statistical interaction graph from high-throughput omics data. These dedicated methods work well even for the case when the number of variables exceeds the number of samples. In this study, we investigate how the graphs extracted from covariance and concentration matrix estimates are related by using Neumann series and transitive closure and through discussing concrete small examples. Considering the ideal case where the true graph is available, we also compare correlation and partial correlation methods for large realistic graphs. In particular, we perform the comparisons with optimally selected parameters based on the true underlying graph and with data-driven approaches where the parameters are directly estimated from the data.

**Electronic supplementary material:**

The online version of this article (doi:10.1186/s13637-016-0052-y) contains supplementary material, which is available to authorized users.

## Introduction

Inference of biological networks including gene regulatory, metabolic, and protein-protein interaction networks has received much attention recently. With the development of high-throughput technologies, it became possible to measure a large number of genes and proteins at once and this led to a challenge to infer a large-scale gene regulatory and protein-protein interaction networks from high-dimensional data [1, 2]. In order to address this challenge, a wide range of network inference methods have been developed such as methods based on correlation or concentration matrices, mutual information, Bayesian networks, ordinary differential equations (ODEs), and Boolean logic [3, 4]. In addition, high-throughput experiments still remain to be costly, and therefore, experiments are usually carried out for a setting with many more genes or proteins than samples. Traditional statistical methods are usually ill-posed in this small *n* large *p* scenario, and novel methods from high-dimensional statistics that assume further structure, such as sparsity, are a good choice for graph reconstruction in this scenario [5]. Correlation methods that are based on the covariance matrix estimation are widely used in reconstructing gene co-expression and module graphs, especially in large-scale biomedical applications [6–8]. However, the edges of the interaction graph resulting from correlation methods include indirect dependencies due to transitive nature of interactions. Accordingly, the effect of indirect edges is getting more dramatic as the graph size grows, and this leads to an inaccurate graph reconstruction. In contrast, methods based on the concentration or partial correlation matrix allow to infer only direct dependencies between variables. In this respect, one can differentiate two graph types resulting from correlation and partial correlation-based methods which we will call covariance and concentration graphs on the following, respectively. Despite the fact that the covariance graph includes indirect dependencies, it is widely used in applications to represent sparse biological graphs by performing simple hard-thresholding [6] or through estimating the covariance matrix with shrinkage methods [9].

The aim of the paper is to shed light on the relation between covariance and concentration graphs and how this relation can be exploited to study the performance of correlation and partial correlation-based methods. In this manuscript, we provide a practical guide for researchers when using correlation and partial correlation methods and we believe that understanding these two concepts allows for a better selection of methods for graph reconstruction problems from high-throughput biological data.

In particular, we discuss different scenarios using simple examples when it is possible to eliminate indirect dependencies in the covariance graph by hard-thresholding and when it is not. Furthermore, we review recent methods that address the problem of direct and indirect dependencies in reconstructed graphs [10, 11] and provide new insights into those methods, both analytically and numerically. Moreover, we perform in silico comparison of two correlation-based and three partial correlation methods on different graph topologies in the high-dimensional case under the setting when the number of variables *p* exceeds the sample size *n*. The selected methods are popular approaches that are widely used in reconstructing large-scale gene regulatory and protein-protein interaction graphs. The first correlation method is based on the sample covariance matrix estimation where one applies hard-thresholding on the entries of sample covariance matrix to eliminate indirect edges in the covariance graph [12]. The second method estimates a sparse version of the covariance matrix via a shrinkage approach [9]. The partial correlation methods that we consider are the nodewise regression method [13], where partial correlations are computed via linear regression, the graphical Lasso method [14] which reconstructs a concentration graph by directly solving for the sparse version of the concentration matrix and an adaptive version of nodewise regression which determines the concentration graph in a two-stage procedure.

## Notation and preliminaries

In the following, we define general notations and symbols which will be used throughout the manuscript. Consider the *p*-dimensional multivariate normally distributed random vector

1 $$ X = (X_{1}, \ldots, X_{p})^{T} \sim \mathcal{N}_{p}(0,\boldsymbol{\Sigma})  $$

with mean zero and covariance ***Σ***. We assume *n* i.i.d. observations of *X* which are given in terms of the *n*×*p* matrix **X**=(**X**_1_,…,**X**_*p*_), where **X**_*i*_ is *n*×1 vector with *i*=1,…,*p*. Then, the sample covariance matrix reads

2 $$ \textbf{S} = \frac{1}{n}{\textbf{X}}^{T}{\textbf{X}}.  $$

Reconstructed and true graphs are written in terms of a undirected graph *G*=(*Γ*,*E*), with *Γ*={1,…,*p*} the set of variables or nodes and *E*⊆*Γ*×*Γ* is a set of edges. Sometimes, we will also deal with weighted graphs where we extend *G* to contain a weight function $w\,: E \rightarrow \mathbb {R}$, such that *w*_*ij*_ denotes the weight of the edge (*i*,*j*)∈*E*. In this paper, we will consider two types of graphs.

1. Covariance graph. The graph in this case is based on the covariance matrix ***Σ***, and the zero entries of the covariance matrix *Σ*_*ij*_=0 indicate that the nodes *i* and *j* are independent [15]. More generally, in terms of probability distributions, we have

$$X_{i} \perp\!\!\!\perp X_{j} \Leftrightarrow p(X_{i},X_{j})=p(X_{i})p(X_{j}). $$

We denote the covariance graph as $\tilde {G}=(\Gamma,\tilde {E})$, accordingly. There is an edge between any two nodes *i* and *j* if *Σ*_*ij*_≠0 and no edge if *Σ*_*ij*_=0. This type of graphs is popular in genomics (for more information, see [16]).

2. Concentration graph. The graph is based on the concentration matrix or inverse covariance matrix ***Θ***≡***Σ*** ^−1^, and zero entries of the concentration matrix *Θ*_*ij*_=0 indicate that any nodes *i* and *j* are conditionally independent given the other nodes. In terms of probability distributions, for arbitrary $k \in \mathcal {N}, k \neq i, j$ it means

$$\begin{aligned} & X_{i} \perp\!\!\!\perp X_{j} |X_{k} \Leftrightarrow p(X_{i}|X_{j},X_{k}) = p(X_{i}| X_{k}) \ \text{or} \\ & X_{i} \perp\!\!\!\perp X_{j} |X_{k} \Leftrightarrow p(X_{i},X_{j}|X_{k}) = p(X_{i}| X_{k})p(X_{j}| X_{k}) \end{aligned} $$

Non-zero entries of the concentration matrix correspond to partial correlations *ρ*_*ij*_ through the relation

3 $$ \rho_{ij} = -\frac{\Theta_{ij}}{\sqrt{\Theta_{ii}\Theta_{jj}}},  $$

for *i*≠*j* and *ρ*_*ij*_=1 for *i*=*j*. There is an edge in the concentration graph between nodes *i* and *j* if *ρ*_*ij*_≠0 and no edge if *ρ*_*ij*_=0 (equivalently for *Θ*_*ij*_). Hence, the concentration graph is equivalent in topology to the graph defining the probabilistic graphical model for the Gaussian case and coincides with the graph defining the associated Gaussian Markov random field. Throughout this paper, we will assume that the true interaction graph corresponds to the concentration graph and therefore refer to it as *G*=(*Γ*,*E*).

In the following, we give a definition of direct and indirect edges in the covariance graph which will be convenient throughout the paper.

### **Definition 1**

Let’s denote the sets of direct and indirect edges in the covariance graph $\tilde {G}$ as $\tilde {E}'$ and $\tilde {E}''$, respectively, with $\tilde {E}=\tilde {E}' \cup \tilde {E}''$. The set of direct edges is then defined as $\tilde {E}'=E$ whereas the set of indirect edges is defined as $\tilde {E}''=\tilde {E} \setminus E$.

## How are covariance and concentration graphs related?

In this section, we will discuss the relationship between covariance and concentration graphs. In particular, we will discuss how to estimate the covariance graph, when the concentration graph is known. We first start by giving some facts about graphical Gaussian models [17].

Let *X*_*d*_, *d*=1,…,*n* be independent samples of $\mathcal {N}(\mu, \boldsymbol {\Sigma })$. The log-likelihood function of the observation *X*_*d*_ is given by

$${} \begin{aligned} L(\mu, \boldsymbol{\Sigma}) = &-\frac{n}{2}\log\det \boldsymbol{\Sigma} - \frac{1}{2}\sum_{d=1}^{n}(X_{d}-\mu)^{T}\boldsymbol{\Sigma}^{-1}(X_{d}-\mu) \\ &= \frac{n}{2}(-\log\det \boldsymbol{\Sigma} - \text{tr} (\boldsymbol{\Sigma}^{-1}\boldsymbol{S})-\\ &-(\bar{X}-\mu)^{T}\boldsymbol{\Sigma}^{-1}(\bar{X}-\mu)), \end{aligned} $$

where $\bar {X}$ represents the sample mean and ***S*** represents the sample covariance matrix. It is then possible to uniquely estimate the mean *μ* and the covariance matrix ***Σ*** using *Θ*_*ij*_=0 as a constraint. Let *C*⊂*Γ* be a clique of the graph *G* that represents a maximal subset of nodes in the graph, such that every node of the set is connected to every other node. Denote ***S***_*C*_ as the submatrix of ***S*** corresponding to that clique. Then, we can recall the following theorem [17].

### **Theorem 1**

If *p*<*n*, then the maximum-likelihood estimator $(\hat {\mu },\hat {\Sigma })$ exists and is determined by (i) $\hat {\mu } = \bar {X}$(ii) (*i*,*j*)∉*E*⇒*Θ*_*ij*_=0,∀*i*,*j*∈*Γ*,*i*≠*j*(iii) $\hat {\boldsymbol {\Sigma }}_{C} = \boldsymbol {S}_{C}$ for all cliques *C* in *G*The solution to (*i*)−(*i* *i* *i*) is unique if ***S*** is nonsingular.

Where $\hat {\mu }$ and $\hat {\boldsymbol {\Sigma }}$ represent the estimated mean and the covariance matrix, respectively. The theorem states that there is a unique $\hat {\boldsymbol {\Sigma }}$ which shares the same elements with ***S*** for the index pairs (*i*,*j*) which are non-zero and satisfy the constraint *Θ*_*ij*_=0. For example, let us consider a simple graph with three nodes, *p*=3, *X*=(*X*_1_,*X*_2_,*X*_3_)^*T*^, where *X*_1_ ╨*X*_3_|*X*_2_ which implies *Θ*_13_=0. In matrix form, this gives

$$\boldsymbol{\Theta}=\left(\begin{array}{ccc} \times & \times & 0 \\ \times & \times & \times \\ 0 & \times & \times \end{array} \right) $$

where (×) represents non-zero entries. According to Theorem 1, the maximum likelihood estimator is given as $\hat {\mu } = \bar {\mu }$ and

$$\hat{\boldsymbol{\Sigma}}=\left(\begin{array}{ccc} s_{11} & s_{12} & \times \\ s_{21} & s_{22} & s_{23} \\ \times & s_{32} & s_{33} \end{array} \right), $$

where (×) for this case computes to *s*_12_ *s*_23_/*s*_22_.

From this result, one can see that all elements of $\hat {\boldsymbol {\Sigma }}$ are determined by entries of sample covariance matrix ***S***. Except $\hat {\Sigma }_{13}$ and $\hat {\Sigma }_{31}$, all elements are the same as in ***S***. This is a nice result from maximum likelihood estimation but it works only in the regime *p*<*n*, where the sample covariance matrix ***S*** is non-singular.

The relationship between the concentration and covariance graphs can be understood by the transitive closure operation [18] which we define in the following way. First, we give a definition for a path.

### **Definition 2**

For a weighted graph *G*=(*Γ*,*E*,*w*) with weight function $w:E \rightarrow \mathbb {R}$, a path *σ* between nodes *i* and *j* is an ordered sequence of 2-tuples of the form *σ*=((*i*,*k*_1_),(*k*_1_,*k*_2_),…,(*k*_*m*_,*j*))∈*P*_*m*_⊆*E* ^*m*^. We call *m* the length of the path and define $w^{\sigma }_{ij} = w_{ik_{1}}w_{k_{1}k_{2}} \cdots w_{k_{m} j}$ as the path weight.

With that, we define the transitive closure as follows.

### **Definition 3**

The transitive closure of a weighted graph *G*=(*Γ*,*E*,*w*) is a weighted graph *G* ^∗^=(*Γ*,*E* ^∗^,*w* ^∗^), with (*i*,*j*)∈*E* ^∗^ iff there exists a path *σ*∈*P*_*m*_ from *i* to *j* in *G* for some $m\in \mathbb {N}$ and with edge weights $w^{*}_{ij} = \sum _{\sigma \in P(i,j)}w^{\sigma }_{ij}$, where *P*(*i*,*j*) is the set of all distinct paths connecting (*i*,*j*) in *G* of any length $m\in \mathbb {N}$.

**Fig. 1:**
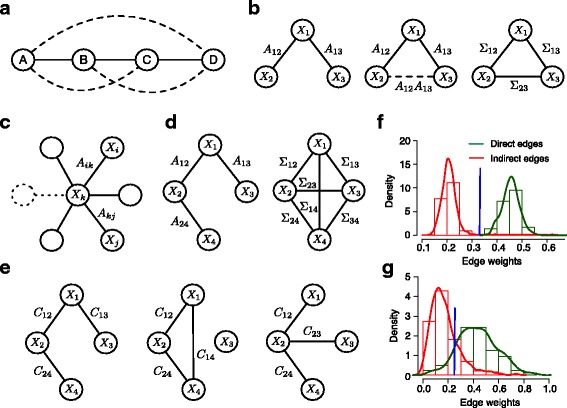
**a** Transitive closure of a graph with four nodes. *Solid edges* indicate existing or direct edges in the graph, whereas *dashed edges* indicate indirect edges which are added to the graph as the result of the transitive closure effect. **b** Three-dimensional true graph (*left*), the transitive closure of the true graph (*middle*), and the corresponding covariance graph constructed from the covariance matrix (*right*). **c** The illustration of a star graph. **d** (*left*) The true example graph which corresponds to the concentration graph, *G*, and (*right*) the covariance graph, $\tilde {G}$ constructed from the covariance matrix. The true graph is sparse, and the covariance graph is fully connected. **e** The covariance graph, $\tilde {G}$ with edge weights given by the correlation matrix *C* (the graph is predicted by thresholding the correlation matrix). (*left*) The graph structure when the condition (A.11) holds (see Additional file 1). (*right*) The graph structure when (A.12) holds (see Additional file 1). Distribution of direct and indirect edges of the covariance graph (*p*=500), when **f** $A_{i(i+1)} \sim \mathcal {N}(0.4,0.0005), \ i=1,\ldots, p-1$ and **g** $A_{i(i+1)} \sim \mathcal {N}(0.4,0.5), \ i=1,\ldots, p-1$. *Vertical line* (*blue*) indicates the optimal threshold that separates two distributions (For more information about **e**, **f**, and **g**, see the text in the Additional file 1)

We associate to *G* and *G* ^∗^ their weighted adjacency matrices denoted ***A*** and ***A*** ^∗^, respectively. Observe that *G* ^∗^ contains self-loops or cycles (e.g., for a node *i* with at least one edge, *i* is connected to *i* by a path of length two through *i*→*j*→*i*), and hence, ***A*** ^∗^ will have non-zero diagonal entries. The transitive closure of the graph is depicted in Fig. 1 a for illustration.

Subsequently, we use the example graph depicted in Fig. 1 b.

It is a simple graph with three nodes, *Γ*={*X*_1_,*X*_2_,*X*_3_} and with the edge set *E*={(*X*_1_,*X*_2_),(*X*_1_,*X*_3_)}. We assume that this graph is weighted and edge weights are given by *A*_12_ and *A*_13_ (Fig. 1 b (left)). The adjacency matrix of *G* then reads

4 $$ \boldsymbol{A}=\left(\begin{array}{ccc} 0 & A_{12} & A_{13} \\ A_{12} & 0 & 0 \\ A_{13} & 0 & 0 \end{array} \right).  $$

We remark that the adjacency matrix (4) is not invertible and generally sparse.

Observing (3), we can construct, without loss of generality, from ***A*** a partial correlation matrix of the form

5 $$ \boldsymbol{\rho} = \boldsymbol{I}+\boldsymbol{A} \quad \text{and hence} \quad \boldsymbol{\Theta} = \boldsymbol{D}(\boldsymbol{I} - \boldsymbol{A})\boldsymbol{D},  $$

where ***D*** is a diagonal scaling matrix to be chosen to determine the diagonal elements of ***Θ***, i.e., $\Theta _{ii} = D^{2}_{ii}$ or $D_{ii} = \sqrt {\Theta _{ii}}$. Naturally, under the performed column and row scaling, ***Θ*** inherits the zero patterns of ***A*** determined by *G*. Moreover, we have

6 $$ \boldsymbol{\Sigma} = \boldsymbol{D}^{-1}(\boldsymbol{I}-\boldsymbol{A})^{-1}\boldsymbol{D}^{-1}  $$

that can be cast into

7 $$ \boldsymbol{\Sigma} = \boldsymbol{D}^{-1}(\boldsymbol{I} + \boldsymbol{A}+ \boldsymbol{A}^{2}+ \boldsymbol{A}^{3} + \cdots)\boldsymbol{D}^{-1}   $$

using the Neumann series, which is convergent for ||***A***||<1. Denoting by *σ*(***A***), the spectral radius of ***A***, then through Gelfand’s theorem by which there exists a *k*>0 such that ||***A*** ^*k*^||<1 if *σ*(***A***)<1, the series more generally converges for *σ*(***A***)<1. We now recall from graph theory that ***A*** ^2^ can be seen as an adjacency matrix of a new graph constructed from *G* by connecting nodes that can be reached by a path of length two in *G*. Generally, entry (*i*,*j*) in ***A*** ^*m*^ will be non-zero if there is a path of length *m* in *G* connecting (*i*,*j*), where we observe that the diagonal elements of ***A*** ^*m*^ need not be zero anymore, due to the presence of possible cycles of length *m* in *G*. The value at entry (*i*,*j*) of ***A*** ^*m*^ or the weight of edge (*i*,*j*) is then the product of weights along one path in *G* and then summed over all the paths connecting (*i*,*j*). Accordingly, the convergent infinite sum

8 $$ \sum_{m=1}^{\infty}\boldsymbol{A}^{m} = (\boldsymbol{I}-\boldsymbol{A})^{-1}-\boldsymbol{I} = \boldsymbol{A}(\boldsymbol{I}-\boldsymbol{A})^{-1}  $$

yields an adjacency matrix of a graph that contains an edge between (*i*,*j*) if there exists a path of any length (*i*,*j*) in *G*. The graph associated with this infinite sum coincides with *G* ^∗^, the transitive closure of *G*, i.e., $\boldsymbol {A}^{*} = \sum _{m=1}^{\infty }\boldsymbol {A}^{m}$ and hence

9 $$ \boldsymbol{\Sigma} = \boldsymbol{D}^{-1}(\boldsymbol{I} + \boldsymbol{A}^{*}) \boldsymbol{D}^{-1}.  $$

The following observations are then immediate. Not-connected subgraphs (disjoint) in the concentration graph *G* transform to not-connected components in the covariance graph. Moreover, taking aside potential cancelation of weights, the subgraphs in *G* ^∗^ are dense, i.e., are fully connected. Using this infinite sum, we show that for special graphs, it is easy to compute single entries of ***Σ*** from the adjacency matrix ***A*** without complete matrix inversion. Generally, the diagonal entries of the concentration matrix ***Θ*** are distinct, and therefore, we assume ***D*** in the example to be

$$\boldsymbol{D}=\left(\begin{array}{ccc} d_{1} & 0 & 0 \\ 0 & d_{2} & 0 \\ 0 & 0 & d_{3} \end{array} \right). $$

We start first with the entry *Σ*_12_=*Σ*_21_ representing the direct edge in the covariance graph. It is possible to represent the corresponding entry in terms of infinite sums by

10 $$ \begin{aligned} \Sigma_{12}=\frac{1}{d_{1}d_{2}}(A_{12}&+A_{12}^{3}+A_{12}A_{13}^{2} + A_{12}^{5} + 2A_{12}^{3}A_{13}^{2} \\ &+A_{12}A_{13}^{4} + A_{12}^{7} + 3A_{12}^{5}A_{13}^{2}\\ &+ 3A_{12}^{3}A_{13}^{4}+A_{12}A_{13}^{6} + \ldots). \end{aligned}  $$

This infinite sum represents geometric series and is convergent. We then multiply this infinite sum with $(A_{12}^{2}+A_{13}^{2})$ and compute the following difference which simplifies to

11 $$ \Sigma_{12} - (A_{12}^{2}+A_{13}^{2})\Sigma_{12}= \frac{A_{12}}{d_{1}d_{2}}.  $$

Dividing both sides of the equality by $(1-A_{12}^{2}-A_{13}^{2})$ gives

12 $$ \Sigma_{12}= \frac{A_{12}}{d_{1}d_{2}(1-A_{12}^{2}-A_{13}^{2})}.  $$

The right hand side of (12) can be expressed with the corresponding entry of the adjacency matrix of the transitive closure graph

13 $$ \Sigma_{12}= \frac{A_{12}^{*}}{d_{1}d_{2}}.  $$

Using the same approach for the entry *Σ*_23_=*Σ*_32_ yields

14 $$ \Sigma_{23}= \frac{A_{12}A_{13}}{d_{2}d_{3}(1-A_{12}^{2}-A_{13}^{2})} = \frac{A_{23}^{*}}{d_{2}d_{3}}.  $$

The same approach holds for diagonal elements as all entries of the covariance matrix have the same denominator $(1-A_{12}^{2}-A_{13}^{2})$.

The covariance matrix is then given by

15 $$ \boldsymbol{\Sigma}=\frac{1}{Z}\left(\begin{array}{ccc} \frac{1}{{d_{1}^{2}}} & \frac{A_{12}}{d_{1}d_{2}} & \frac{A_{13}}{d_{1}d_{3}} \\ \frac{A_{12}}{d_{1}d_{2}} & \frac{1-A_{13}^{2}}{{d_{2}^{2}}} & \frac{A_{12}A_{13}}{d_{2}d_{3}} \\ \frac{A_{13}}{d_{1}d_{3}} & \frac{A_{12}A_{13}}{d_{2}d_{3}} & \frac{1-A_{12}^{2}}{{d_{3}^{2}}} \end{array} \right),  $$

where $Z =1-A_{12}^{2}-A_{13}^{2}$.

Equivalently,

16 $$\begin{array}{*{20}l} \boldsymbol{\Sigma} &=\left(\begin{array}{ccc} \frac{1 + A^{*}_{11}}{{d_{1}^{2}}} & \frac{A_{12}^{*}}{d_{1}d_{2}} & \frac{A_{13}^{*}}{d_{1}d_{3}} \\ \frac{A_{12}^{*}}{d_{1}d_{2}} & \frac{1 + A^{*}_{22}}{{d_{2}^{2}}} & \frac{A_{23}^{*}}{d_{2}d_{3}} \\ \frac{A_{13}^{*}}{d_{1}d_{3}} & \frac{A_{23}^{*}}{d_{2}d_{3}} & \frac{1 + A^{*}_{33}}{{d_{3}^{2}}}\end{array} \right) \\ & = \boldsymbol{D}^{-1}(\boldsymbol{I} + \boldsymbol{A}^{*})\boldsymbol{D}^{-1}.  \end{array} $$

To sum up, the entries of the covariance matrix can be obtained by applying the transitive closure from Definition 3 on the concentration graph in addition to a general scaling through ***D***. Interestingly, for particular graphs, as the example above, more structure of the concentration graph can be exploited for computing the transitive closure and hence the covariance matrix.

For instance, the following result provides the expressions of the transitive closure for a star graph Fig. 1 c.

### **Proposition 1**

Consider a star graph with |*Γ*|=*p*, |*E*|=*p*−1 and adjacency matrix ***A***. Denote the index of the hub node of the star by *k* and define $c = 1-\sum _{l=1}^{p} A_{kl}A_{lk}$, then ∀*i*≠*k* and ∀*j*≠*k* we have $A^{*}_{ij} = A_{ik}A_{kj}/c$, $A^{*}_{ik} = A_{ik}/c$, and $A^{*}_{kk} = 1/c-1$.

The proof of Proposition 1 is given in Additional file 1. The result moreover indicates that the entries of the transitive closure matrix ***A*** ^∗^ could be related to each other. A simple relation can be obtained by considering the correlation matrix, i.e., the normalized version of the covariance matrix

***C***=***Λ*** ^−1^ ***Σ*** ***Λ*** ^−1^

with diagonal scaling matrix ***Λ*** with elements $\Lambda _{ii} = \sqrt {\Sigma _{ii}}$. In order to formalize the relation, we introduce the following variant of transitive closure.

### **Definition 4**

The minimal transitive closure *T* of a weighted graph *G*=(*Γ*,*E*,*w*), *G*↦*T*(*G*) is the weighted graph $\tilde {G}=(\Gamma,\tilde {E},\tilde {w})$ with $(i,j) \in \tilde {E}$ iff there exists a path between (*i*,*j*) with edge weights $\tilde {w}_{ij} = \sum _{\sigma \in \tilde {P}(i,j)}w^{\sigma }_{ij}$ where $\tilde {P}(i,j)$ is the set of distinct paths *σ*_*ij*_ that are of minimal length.

With that, we have the following.

### **Proposition 2**

Consider a concentration graph that is a star graph *G*=(*Γ*,*E*,*w*) and denote its associated covariance graph as *G* ^′^=(*Γ* ^′^,*E* ^′^,*w* ^′^), with weights *w* ^′^ corresponding to the correlation coefficients. Defining the graph $\hat {G} = (\Gamma,E,\hat {w})$ with $\hat {w}_{ij} = w'_{ij}$ for all (*i*,*j*)∈*E*, then it holds that $T(\hat {G}) = G'$.

The proof of Proposition 2 is given in Additional file 1. This proposition indicates that the covariance graph with weights from the correlation matrix is the minimal transitive closure of the concentration graph with weights given by the correlation matrix, i.e., indirect edge weights can be obtained by closure on the direct edges.

In the following, we demonstrate an application of Proposition 2 for our running example. A diagonal scaling matrix for this example ***Λ*** computes to

$$\boldsymbol{\Lambda}=\frac{1}{\sqrt{Z}}\left(\begin{array}{ccc} \frac{1}{d_{1}} & 0 &0 \\ 0 & \frac{\sqrt{1-A_{13}^{2}}}{d_{2}} & 0 \\ 0 & 0 & \frac{\sqrt{1-A_{12}^{2}}}{d_{3}}\end{array} \right), $$

where $Z =1-A_{12}^{2}-A_{13}^{2}$. Then, we calculate the correlation matrix

$$\boldsymbol{C}=\left(\begin{array}{ccc} 1 & \frac{A_{12}}{\sqrt{1-A_{13}^{2}}} & \frac{A_{13}}{\sqrt{1-A_{12}^{2}}} \\ \frac{A_{12}}{\sqrt{1-A_{13}^{2}}} & 1 & \frac{A_{12}A_{13}}{\sqrt{f(A_{12},A_{13})}} \\ \frac{A_{13}}{\sqrt{1-A_{12}^{2}}} & \frac{A_{12}A_{13}}{\sqrt{f(A_{12},A_{13})}} & 1 \end{array} \right),$$

where $f(A_{12},A_{13})=(1-A_{12}^{2})(1-A_{13}^{2})$.

Here, the edge weights of the covariance graph are defined in terms of the edge weights of the concentration graph

17 $$ \begin{aligned} \tilde{A}_{1} = &\frac{A_{12}}{\sqrt{1-A_{13}^{2}}}, \ \tilde{A}_{2} = \frac{A_{13}}{\sqrt{1-A_{12}^{2}}} \\ & \tilde{A}_{3} = \frac{A_{12}A_{13}}{\sqrt{(1-A_{12}^{2})(1-A_{13}^{2})}}. \end{aligned}  $$

We observe that the exact relation holds $\tilde {A}_{3}=\tilde {A}_{1}\tilde {A}_{2}$, and the covariance graph can be regarded as the transitive closure of the concentration graph with edge weights $\tilde {A}_{1}$ and $\tilde {A}_{2}$.

Further examples of the set of graph for which this relation holds are chain graphs and tree graphs, which are numerically shown in our study.

### Estimating sparse covariance graph via hard-thresholding the covariance matrix

After establishing a link between concentration and covariance graphs, we discuss how to obtain a sparse covariance graph by performing hard-thresholding on the entries of the covariance matrix with concrete examples that are given in Fig. 1 d, e. Here, our goal is to examine when it is possible to get the covariance graph which is similar to the concentration graph in terms of non-zero edges after hard-thresholding is applied. In particular, we give simple conditions on the entries of an adjacency matrix that allow the covariance graph to preserve a corresponding set of edges as in the concentration graph. A detailed description of this section is given in Additional file 1.

### Graph reconstruction via network deconvolution

As we stated earlier, the concentration and covariance graphs can be related via the Neumann series. In the following, we briefly review a network deconvolution approach by Feizi et al. [10], which is based on a similar idea. A closely related method, called network silencing, is proposed in [11]. Strictly speaking, both methods are only applicable in the setting *p*<*n*.

For an unknown adjacency matrix ***A***, [10] assume to be given a so-called observation matrix ***Σ***_*M*_ related to ***A*** through

18 $$ \boldsymbol{\Sigma}_{M} = \boldsymbol{A}(\boldsymbol{I}-\boldsymbol{A})^{-1}=\boldsymbol{A} + \boldsymbol{A}^{2}+ \boldsymbol{A}^{3}+ \ldots +,  $$

which coincides with our definition of a transitive closure of ***A*** in (8). For many applications considered in [10], the observation matrix is taken to be the covariance or correlation matrix computed from experimental data. Comparing (18) with (6) indicates that the assumed form of the observation matrix does not cover the general form for covariance or correlation matrices.

The authors then solve for ***A*** in (18) to obtain

19 $$ \boldsymbol{A} = \boldsymbol{\Sigma}_{M}(\boldsymbol{I}+\boldsymbol{\Sigma}_{M})^{-1},  $$

which was coined network deconvolution and aims to recover the graph of direct edges. Observing (9) indicates that the rank deficiency of a covariance matrix obtained from *n*<*p* samples also implies a rank deficiency of (***I***+***A*** ^∗^) which is the matrix to be inverted in network deconvolution according to (19). Hence, deconvolution cannot be applied directly for *p*>*n* unless one applies regularization, for instance, through hard-thresholding [19]. Contrasting the definition (18) of ***Σ***_*M*_ given in [10], the authors finally use a modified version where the diagonal elements are set to zero leading to an inconsistency in the definition of the deconvolution (19). As discussed earlier, the transitive closure (18) has indeed non-zero diagonal entries due to cyclic paths made possible through higher order terms. Consequently, redefining ***Σ***_*M*_=***A*** ^∗^−***V***, with a diagonal matrix ***V***=diag(***A*** ^∗^), the exact network deconvolution for the adapted transitive closure would read

20 $$ \boldsymbol{A} = \boldsymbol{\Sigma}_{M}(\boldsymbol{I}+ \boldsymbol{V} + \boldsymbol{\Sigma}_{M})^{-1} + \boldsymbol{V}(\boldsymbol{I}+ \boldsymbol{V} + \boldsymbol{\Sigma}_{M})^{-1}.  $$

However, resorting to the Neumann series again, we see that the zero patterns of (20) and (19) coincide, and hence, this adaptation does not affect the obtained the graph structure. Subsequently, we consider the scaled version of network deconvolution which is mainly used in [10]

21 $$ \boldsymbol{\tilde{A}} = \alpha\boldsymbol{\Sigma}_{M}(I+\alpha\boldsymbol{\Sigma}_{M})^{-1},  $$

where *α* is a scaling parameter that should control the convergence of the matrix inversion in (19).

Although the expression (19) is general, [10] state that a necessary assumption of network deconvolution is that indirect edge weights encoded in ***Σ***_*M*_ can be expressed as a product of direct edge weights along the path according to ***A***. However, it is not clear which type of graphs ***A*** give rise to such a weight relation in the observation matrix (e.g., see Proposition 2 and its discussion). In the following, we demonstrate that such a relation holds for chain graphs for any *α*.

#### Network deconvolution for chain graphs

We first start with a small case study and further generalize it to arbitrary dimensions. Consider a four-node graph given in Fig. 1 d (right) which contains six edges, out of which three are indirect ones. For simplicity, we assume that direct edges are given by *θ*=*Σ*_12_=*Σ*_13_=*Σ*_24_ and that second-order and third-order edges are *s*_1_=*Σ*_14_=*Σ*_23_ and *s*_2_=*Σ*_34_, respectively. We then get the following observation matrix representing the covariance graph

22 $$ \boldsymbol{\Sigma}_{M}=\left(\begin{array}{cccc} 0 & \theta & s_{1} & s_{2} \\ \theta & 0 & \theta & s_{1} \\ s_{1} & \theta & 0 & \theta \\ s_{2} & s_{1} & \theta & 0 \end{array} \right).  $$

Following the assumptions in [10], we investigate how the indirect and direct edges have to be related for a given *α* such that deconvolution is exact. Therefore, we compute (21) and determine when indirect weights in $\boldsymbol {\tilde {A}}$ are zero. It corresponds to solving a system of two equations for the indirect edges *s*_1_ and *s*_2_

$$\begin{array}{@{}rcl@{}} \theta^{3}\alpha^{2}-s_{2}\theta^{2}\alpha^{2} + {s_{1}^{2}}\theta \alpha^{2} - 2s_{1}\alpha + s_{2}=0 \\ -\theta^{2}\alpha -s_{2}\theta\alpha+{s_{1}^{2}}\alpha + s_{1}=0. \end{array} $$

Alternatively, one can see that for general *s*_1_ and *s*_2_, there exists no single scaling parameter *α* that satifies both equations. For *s*_1_ and *s*_2_, we then get the following solutions

23 $$ s_{1,1}= \frac{2\theta^{2}\alpha^{2}-1}{\alpha} \ \text{and} \ s_{1,2}=\alpha\theta^{2}  $$

24 $$ s_{2,1}=4\theta^{3}\alpha^{2}-3\theta \ \text{and} \ s_{2,2}=\alpha^{2}\theta^{3}.  $$

Considering the second solutions *s*_1,2_=*α* *θ* ^2^ and *s*_2,2_=*α* ^2^ *θ* ^3^, one finds that indirect edge weights are indeed the product of direct edges along the path.

One can intuitively extend this relation to higher-order indirect edges as a network size grows as(*α* ^3^ *θ* ^3^,*α* ^4^ *θ* ^5^,…,*α* ^*p*−2^ *θ* ^*p*−1^) where *p* is the number of variables.

We rewrite this relation in a compact form

25 $$ S_{k} = \alpha^{k-1}\theta^{k}, \ k=2,\ldots, p-1,  $$

where *S*_*k*_ represents indirect edges of *k*-th order.

In the following, we show what happens when the relation (25) holds. We therefore define the general observation matrix using (25) as

$$\boldsymbol{\Sigma}_{M} = \left(\begin{array}{ccccc} 0 & \theta & \alpha\theta^{2} & \ldots & \alpha^{p-2}\theta^{p-1} \\ \theta & 0 & \theta & \ldots & \alpha^{p-3}\theta^{p-2} \\ \alpha\theta^{2} & \theta & 0 & \ldots & \alpha^{p-4}\theta^{p-3} \\ \vdots & \vdots & \vdots & \ddots & \vdots \\ \alpha^{p-2}\theta^{p-1} & \alpha^{p-3}\theta^{p-2} & \times & \ldots & 0 \end{array} \right). $$

For (21), we then calculate ***B***=***I***+*α* ***Σ***_*M*_, that is

$$\boldsymbol{B}= \left(\begin{array}{ccccc} 1 & \alpha\theta & \alpha^{2}\theta^{2} & \ldots & \alpha^{p-1}\theta^{p-1} \\ \alpha\theta & 1 & \alpha\theta & \ldots & \alpha^{p-2}\theta^{p-2} \\ \alpha^{2}\theta^{2} & \alpha\theta & 1 & \ldots & \alpha^{p-3}\theta^{p-3} \\ \vdots & \vdots & \vdots & \ddots & \vdots \\ \alpha^{p-1}\theta^{p-1} & \alpha^{p-2}\theta^{p-2} & \times & \ldots & 1 \end{array} \right), $$

which is known as the Kac-Murdock-Szëgo matrix, i.e., a symmetric Toeplitz matrix [20, 21] with elements

26 $$ B_{ij}= (\alpha\theta)^{|i-j|}, \ |\theta|<1, \ i,j=1,\ldots, p.  $$

This matrix has a simple tridiagonal inverse

$$\boldsymbol{B}^{-1}= W \left(\begin{array}{ccccc} 1 & -\alpha\theta & 0 & \cdots & 0 \\ -\alpha\theta & 1+\alpha^{2}\theta^{2} & -\alpha\theta & \cdots & 0 \\ \vdots & \ddots & \ddots & \ddots & \vdots \\ 0 & \cdots & -\alpha\theta & 1+\alpha^{2}\theta^{2} & -\alpha\theta \\ 0 & \cdots & 0 & -\alpha\theta & 1 \end{array} \right), $$

where *W*=(1−*α* ^2^ *θ* ^2^)^−1^.

Finally, we calculate the deconvolved adjacency matrix $\boldsymbol {\tilde {A}}=\alpha \boldsymbol {\Sigma }_{M}\boldsymbol {B}^{-1}$ from (21)

$$\boldsymbol{\tilde{A}}= W \left(\begin{array}{ccccc} -\alpha^{2}\theta^{2} & \alpha\theta & 0 & \ldots & 0 \\ \alpha\theta & -2\alpha^{2}\theta^{2} & \alpha\theta & \ldots & 0 \\ \vdots & \ddots & \ddots & \ddots & \vdots \\ 0 & \ldots & \alpha\theta & -2\alpha^{2}\theta^{2} & \alpha\theta \\ 0 & \ldots & 0 & \alpha\theta & -\alpha^{2}\theta^{2} \end{array} \right), $$

which is again a tridiagonal matrix that represents a chain graph. Observation matrices obtained from data will not obey to this specific structure, hence the named product rule does not apply in general.

#### Effect of scaling parameter on the output of network deconvolution

The scaling parameter *α* is introduced in [10] to improve network deconvolution. However, we show with simple examples that particular choices for *α* can lead to unwanted elimination of direct edges. We again consider the four-node graph that contains three direct and three indirect edges which are *θ*_1_,*θ*_2_,*θ*_3_ and *s*_1_,*s*_2_,*s*_3_, respectively. The assignment of direct and indirect edges corresponds a chain graph. The observation matrix is given by

27 $$ \boldsymbol{\Sigma}_{M}=\left(\begin{array}{cccc} 0 & \theta_{1} & s_{1} & s_{2} \\ \theta_{1} & 0 & \theta_{2} & s_{3} \\ s_{1} & \theta_{2} & 0 & \theta_{3} \\ s_{2} & s_{3} & \theta_{3} & 0 \end{array} \right)  $$

We element-wise solve the network deconvolution problem (21) and solve for *α* such that a particular direct edge, i.e., *θ*_1_ in $\boldsymbol {\tilde {A}}$ will be zero. In particular,

28 $$ \begin{aligned} \alpha_{1,2}^{\theta_{1}}&=\frac{\theta_{2}s_{1}+s_{2}s_{3} \pm \sqrt{\Delta_{\theta_{1}}}}{2M_{\theta_{1}}} \\ \Delta_{\theta_{1}}&=(\theta_{2}s_{1}+s_{2}s_{3})^{2}-4\theta_{1}M_{\theta_{1}}\\ M_{\theta_{1}}&=-\theta_{1}{\theta_{3}^{2}} + \theta_{2}\theta_{3}s_{2}+\theta_{3}s_{1}s_{3}. \end{aligned}  $$

It is easy to derive the same for other direct edges. If the scaling parameter is chosen as in (28), then only the direct edge *θ*_1_ will be zero, whereas other edges including indirect edges will be non-zero. In applications, it is difficult to choose the scaling parameter for which network deconvolution discriminates correctly between direct and indirect edges. The user needs to be aware of the fact that for some choices of *α* network, deconvolution can negatively affect the accuracy by removing direct edges instead of indirect ones.

In the following, we investigate how this scaling parameter affects indirect edges of different order with numerical simulations. For this purpose, we choose a six-node chain graph, generate synthetic data using the workflow illustrated in Fig. 4, and compute the correlation matrix. The covariance graph reconstructed from the correlation matrix is accordingly fully connected and has five direct and ten indirect edges, where edges of the same order were assigned the same weight.

To quantify the effect of network deconvolution with different scaling parameters, we measure the discriminative ratio

29 $$ r = \log \frac{\langle A_{ij}^{\text{dir}}\rangle/\langle A_{ij}^{\text{indir}} \rangle}{\langle \Sigma_{M,ij}^{\text{dir}} \rangle/ \langle \Sigma_{M,ij}^{\text{indir}}\rangle},  $$

where $\langle A_{ij}^{\text {dir}}\rangle $ and $\langle \Sigma _{M,ij}^{\text {dir}} \rangle $ are the average weights of direct edges in $\boldsymbol {\tilde {A}}$ and ***Σ***_*M*_, whereas $\langle A_{ij}^{\text {indir}}\rangle $ and $\langle \Sigma _{M,ij}^{\text {indir}} \rangle $ represent the average weights of indirect edges in $\boldsymbol {\tilde {A}}$ and ***Σ***_*M*_, respectively. The average is taken over all edges of the same order. We compute the discriminative ratio for each order separately.

**Fig. 2:**
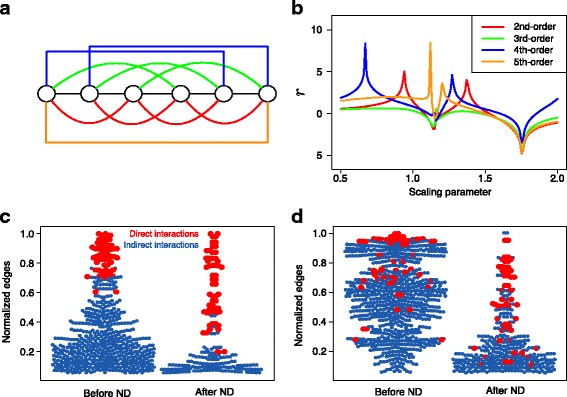
Simulation study for network deconvolution (ND). **a** Illustration of a graph with direct and indirect edges of different order; original graph is a chain graph. **b** Simulations conducted on the graph depicted in (**a**) with different scaling parameters. Shown is the log discriminative ratio given in (29). **c** Effect of network deconvolution on direct and indirect edges. If the indirect edges are clearly separable in the covariance matrix, then network deconvolution can better separate them from direct edges. **d** If direct and indirect edges are not separable in the covariance matrix, then network deconvolution cannot separate them too

A positive log-ratio indicates that network deconvolution can better discriminate direct and indirect edges than in the covariance graph, while a negative log-ratio shows the opposite. For instance, for positive log-ratios, hard-thresholding on the deconvolved matrix would yield more accurate results. However, Fig. 2 b shows that edges of different order are better discriminated at different values of *α*. Thus, the effect of *α* is not uniform for all indirect edges which means that any improved discrimination after deconvolution is due to edges of some order. For example, for *α*∈(0.5,1.5) network, deconvolution better discriminates the second, fourth, and fifth order edges, whereas it fails to discriminate the third order edge. For *α*∈(1.5,2), the method fails to better discriminate any edge. With simulations, we also show that both network deconvolution and network silencing approaches can help better discriminate direct and indirect edges if edges are already separable in the covariance graph as it is shown in Fig. 2 c. If the absolute values of some indirect edges in the covariance graph are larger than the absolute values of direct edges, then both methods fail to discriminate them (Fig. 2 d).

## Methods

In this section, we give a brief overview of methods that are used in our comparison study. For a fair comparison, we select two correlation and three partial correlation-based methods (Table 1). Correlation-based approaches are the thresholded covariance and the covariance Lasso methods [9]. Partial correlation-based approaches are the nodewise regression Lasso [13], the graphical Lasso [14], and the adaptive Lasso. The intuition behind a selection of these methods is their simplicity in terms of free parameters, and all considered methods contain only one free parameter. These parameters are the element-wise thresholding for the thresholded covariance matrix and sparsity inducing penalty parameters for the covariance Lasso, the nodewise regression Lasso, the graphical Lasso, and the adaptive Lasso. Here, Lasso methods are L1-regularization-based approaches, meaning that all include a penalty term ||.||_1_.

**Table 1:** A list of graph reconstruction methods considered in this study

Methods	Category
Thresholded sample covariance [6]	Correlation
Covariance Lasso [9]	Correlation
Nodewise regression Lasso [13]	Partial correlation
Graphical Lasso [14]	Partial correlation
Adaptive Lasso [22]	Partial correlation

### Correlation-based methods

#### Hard-thresholding of sample covariance matrix

**Fig. 3:**
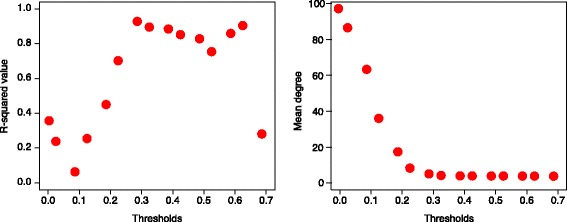
Selecting a hard-threshold based on the *R* ^2^ and the mean degree values which are plotted versus hard-thresholding values. The hard-thresholded values starting from 0.3 give rise to scale-free topology except 0.7 and higher. The corresponding mean degree values are relatively low indicating a sparsity of the underlying graph. The numbers in the plot represent different thresholds which are plotted for illustration purposes

The simplest way to reconstruct the covariance graph is based on the sample covariance matrix which is easy to compute. However, the graph resulting from the sample covariance matrix is fully connected. One way to reconstruct a sparse covariance graph is to threshold the sample covariance matrix. This method is popular in applications; for instance, it is at the core of WGCNA package [6]. One study showed that the connected components of the concentration graph can be completely described by the covariance graph obtained by thresholding the sample covariance matrix [12] (Fig. 3).

However, a selection of the threshold is hard to tackle analytically. Recently, some methods have been developed to choose the threshold from the data [19, 23, 24]. However, these methods have been designed for the case *p*<*n* and do not perform well in the *p*>*n* setting.

Graph reconstruction with thresholding the sample covariance matrix based on the scale-free criteria of the graph is widely used in practice, especially in biomedical applications [7, 25], and often applied in case *p*>*n*. In the following, we are going to briefly review this method. Scale-free graphs are characterized by a power law degree distribution

30 $$ P(k) = bk^{-\gamma},  $$

where *k* is the node degree, *γ* is the degree exponent, and *b* is the normalization constant [26, 27]. Some biological graphs have been reported to exhibit a power law have degree distributions with 2<*γ*<3 [27].

Assume a sample covariance matrix ***S*** defined as in (2). We further define the thresholding operation *T*_*d*_(*S*_*ij*_) yielding sample covariance matrix elements thresholded at *d*. To choose the threshold *d*, we fit an affine function $f(k) = -\hat {\gamma }k + \hat {b}$ to the empirical degree distribution of a graph obtained by thresholding at *d* in the log domain and compute the *R* ^2^ value of the fit (0<*R* ^2^<1) (Fig. 3 (left)). In addition, we also compute mean degrees $\bar {k}=p^{-1}\sum _{i=1}^{p}\tilde {k}_{i}$, where $\tilde {k}_{i}=\sum _{j=1}^{p}T_{d}(S_{ij})$ (Fig. 3 (right)). In particular, we are interested in high *R* ^2^ values and, for sparsity, low mean degree values $\bar {k}$. We also require $\hat {\gamma } > 0$, so that the slope of the fitted linear function is negative. High *R* ^2^, low mean degree values, $\bar {k}$ and $\hat {\gamma } > 0$ give rise to graphs with a few connections and that a few nodes have more connections compared to other nodes. This indicates that the graph obtained from *T*_*d*_(***S***) is approximately scale-free. So far, we have introduced a sparse covariance estimation using hard-thresholding where hard-thresholding is performed after the estimation of the sample covariance matrix. In the following section, we discuss a direct estimation of the sparse covariance matrix in which no hard-thresholding is involved.

#### Covariance Lasso

In this section, we shortly review the sparse covariance matrix estimation introduced in [9] which is called *Covariance Lasso*. In contrast to hard-thresholding introduced in the previous section, the sparsity in the covariance matrix is achieved by minimizing a log-likelihood function of the form

31 $$ L(\boldsymbol{\Sigma}|\boldsymbol{S}) = \log \det \boldsymbol{\Sigma} + \text{tr}(\boldsymbol{\Theta} \boldsymbol{S}) + \lambda_{\text{cov}} ||\boldsymbol{P} \circ \boldsymbol{\Sigma}||_{1},  $$

where ***S*** is the sample covariance matrix as defined in (2) and *λ*_cov_ is the penalty parameter which induces sparsity in off diagonal elements of ***Σ***, whereas ***P*** is a matrix with nonnegative elements and ∘ denotes elementwise multiplication. The matrix ***P*** can be chosen as the matrix of ones or zeros on the diagonal to avoid shrinking diagonal elements of ***Σ***. The objective function given in (31) is nonconvex which is due to the term log det***Σ*** and has several local minima, which makes the optimization problem difficult. Since the objective function contains convex and concave terms, a majorization-minimization approach is used to solve the problem. This approach was successfully applied earlier on similar problems [28, 29]. The concave part of the objective function (31) is approximated by its tangent at ***Σ***_0_

32 $$ \log \det \boldsymbol{\Sigma} \leq \log \det \boldsymbol{\Sigma}_{0} + \text{tr}(\boldsymbol{\Sigma}_{0}(\boldsymbol{\Sigma}-\boldsymbol{\Sigma}_{0})).  $$

Then, the majorized function is convex and given by

33 $$ \begin{aligned} f(\boldsymbol{\Sigma},\boldsymbol{\Sigma}_{0}|\boldsymbol{S}) = &\log \det \boldsymbol{\Sigma}_{0} + \text{tr}(\boldsymbol{\Theta}_{0}\boldsymbol{\Sigma}) - \\ & - \text{tr}(\boldsymbol{\Theta}_{0}\boldsymbol{\Sigma}_{0}) + \text{tr}(\boldsymbol{\Theta} \boldsymbol{S}) + \\ & + \lambda_{\text{cov}} ||\boldsymbol{P} \circ \boldsymbol{\Sigma}||_{1}, \end{aligned}  $$

where ***Σ***_0_=***S*** or ***Σ***_0_=diag(***S***) and $\boldsymbol {\Theta }_{0}=\boldsymbol {\Sigma }_{0}^{-1}$. So one needs to estimate the covariance matrix by

34 $$ \begin{aligned} \boldsymbol{\hat{\Sigma}}=\arg \min_{\boldsymbol{\Sigma} \succ 0} f(\boldsymbol{\Sigma},\boldsymbol{\Sigma}_{0}|\boldsymbol{S}). \end{aligned}  $$

In the case *p*>*n*, the sample covariance matrix ***S*** is not full rank, and to avoid this, one needs to use ***S***=***S***+*s* *I*, for some small regularizing parameter *s*>0.

In applications, the penalty parameter *λ*_cov_ should be determined from the data and *K*-fold cross-validation is used for this purpose. First, the samples (1,…,*n*) which correspond to the rows of the design matrix **X** are partitioned into *K* subsets which are used as training and validation sets. Initially, the covariance matrix is estimated as in (34) using the training set. We denote it as $\boldsymbol {\hat {\Sigma }}_{T}$. The validation set is used to compute the sample covariance matrix, which we denote as ***S***_*V*_. The penalty parameter is then computed via

35 $$ \lambda_{\text{cov}}^{\text{CV}} = \arg \max_{\lambda >0}\bigg\{\frac{1}{K}\sum_{i=1}^{K} L(\boldsymbol{\hat{\Sigma}}_{T}|\boldsymbol{S}_{V})\bigg\},  $$

where $L(\boldsymbol {\hat {\Sigma }}_{T}|\boldsymbol {S}_{V})$ is defined in (31).

### Partial correlation-based methods

#### Nodewise regression Lasso

In this section, we discuss an efficent partial correlation-based method that estimates the concentration graph through independent shrinkage regressions [13]. Accordingly, we assume **X**_*i*_, *i*∈*Γ* to be a response variable and **X** ^∖*i*^ to be the matrix of predictor variables consisting of the remaining *p*−1 variables. In order to get an estimate for the node *i*∈*Γ*, one regresses this node with the remaining nodes *j*∈*Γ*∖{*i*} and get a linear model of the form

36 $$ \mathbf{X}_{i} = \mathbf{X}^{\setminus i}\boldsymbol{\beta}^{i} + \boldsymbol{\epsilon}_{i},  $$

where vector ***β*** ^*i*^ is the set of *p*−1 regression coefficients associated to node *i* and $\mathbb {E}[\boldsymbol {\epsilon }_{i}]=\mathbf {0}$. Denoting an element of vector ***β*** ^*i*^ as the regression coefficient ${\beta ^{i}_{j}}$, with *j*∈*Γ*∖{*i*}, then this coefficient can be related to the concentration matrix as

37 $$ {\beta^{i}_{j}} = \Theta_{ij} / \Theta_{ii} \quad \text{for}\quad j \neq i.  $$

Using (3), it is hence also possible to represent the regression coefficients in terms of partial correlations

38 $$ {\beta^{i}_{j}}= -\rho_{ij} \sqrt{\frac{\Theta_{jj}}{\Theta_{ii}}}.  $$

From this relationship, one can notice that regression coefficients correspond to normalized partial correlations. The regression coefficients from the linear model (36) are estimated via traditional Lasso [30]

39 $$ \hat{\boldsymbol{\beta}}^{i} = \arg\min_{\boldsymbol{\beta}^{i}}\left(\frac{1}{n}||\mathbf{X}_{i} - \mathbf{X}^{\setminus i} \boldsymbol{\beta}^{i}||^{2}_{2} + \lambda_{L}||\boldsymbol{\beta}^{i}||_{1}\right),  $$

where *λ*_*L*_>0 denotes the penalty parameter. In order to estimate a whole graph, this procedure is applied to all nodes, by regressing each node by the remaining nodes. Nodewise regression Lasso returns sparse estimates which are not symmetric. In particular, there are two different estimates for each edge between any two nodes, which are estimated from two different regression problems. To decide for the absence or presence of the corresponding edge in the concentration graph, AND and OR operations are proposed in [13], i.e., an edge (*i*,*j*) is present if $\hat {\beta }^{i}_{j}$ and/or $\hat {\beta }^{j}_{i}$ are non-zero.

#### Graphical Lasso

One way to reconstruct the concentration graph is by directly estimating the concentration matrix which elements correspond to normalized partial correlations which can be seen from (37) and (38). One can estimate the concentration matrix by maximizing the penalized log-likelihood function of the form

40 $$ L(\boldsymbol{\Theta}|\boldsymbol{S}) = \log \det \boldsymbol{\Theta} - \text{tr}(\boldsymbol{S}\boldsymbol{\Theta}) - \lambda_{G}||\boldsymbol{\Theta}||_{1},  $$

where *λ*_*G*_ is the parameter which controls the size of the penalty. This log-likelihood function is convex and can be solved by a block coordinate descent method proposed in [31]. The estimated concentration matrix is symmetric, and there are no additional AND or OR operations needed.

#### Adaptive Lasso

In applications, the penalty parameters *λ*_*L*_ in (39) and *λ*_*G*_ in (40) are chosen by cross-validation. However, a cross-validated choice of these penalty parameters does not lead to a consistent model selection and leads to overestimation [5, 13]. Therefore, it is suggested to apply cross-validation using the adaptive Lasso (adaptive version of nodewise regression) which gives a sparser solution compared to cross-validation with nodewise regression and graphical Lasso. Given the data where the underlying graph is not known, it is challenging to determine a good Lasso penalty from the data. One study showed that it is possible to assign different weights to different coefficients thereby allowing the coefficients to be non-equally penalized in the *L*_1_ penalty [22]. This is achieved by the following estimator:

41 $$ \hat{\boldsymbol{\beta}}^{i} = \arg \min_{\boldsymbol{\beta}^{i}} \left(\frac{1}{n}||\mathbf{X}_{i} - \mathbf{X}^{\setminus i} \boldsymbol{\beta}^{i}||^{2}_{2} + \lambda_{L} \sum_{j \neq i}^{p} \frac{|{\beta^{i}_{j}}|}{|\tilde{\beta}^{i}_{j}|}\right),  $$

where $\tilde {\boldsymbol {\beta }}^{i}$ are initial estimates from (39) and used as weights. It is suggested to estimate $\tilde {\beta }^{i}$ with the penalty parameter computed through cross-validation. In the second step, it is suggested to select the penalty parameter again by cross-validation in the adaptive Lasso. The adaptive Lasso has the property that if the initial estimates $\tilde {\beta }^{i}_{j}=0$, then the final estimates resulting from the adaptive Lasso are also $\hat {\beta }^{i}_{j}=0$. If the initial estimates $\tilde {\beta }^{i}_{j}$ are large, then the adaptive Lasso applies a small penalty for these estimates and vice versa. This way, the adaptive Lasso allows to reduce the number of false positives from the first step and yields a sparse solution.

## Comparison of correlation- and partial correlation-based methods

### Generating synthetic data from different graph topologies

**Fig. 4:**
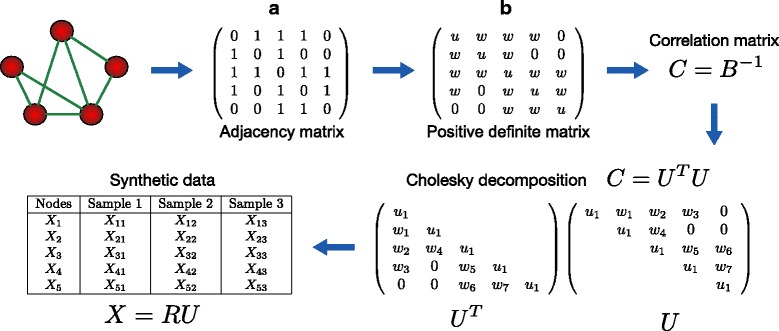
Workflow for generating synthetic data from a given graph topology. Initially, we construct a graph of interest and then build the adjacency matrix ***A*** which elements are ones and zeros. In the next step, we transform ***A*** to the positive definite matrix ***B***. We then take an inverse of the positive definite matrix ***B*** and calculate the correlation matrix ***C***. In the next step, we factorize the correlation matrix using a Cholesky decomposition and obtain an upper triangular matrix ***U***. We then generate a random matrix ***R***, the columns of which are independent and identically distributed from $\mathcal {N}(0,1)$. A row size of ***R*** is equal to a column size of ***U***, and a column size is equal to a sample size that we want to generate. Finally, we multiply ***R*** with ***U*** to get a new data with the sample size of interest

**Fig. 5:**
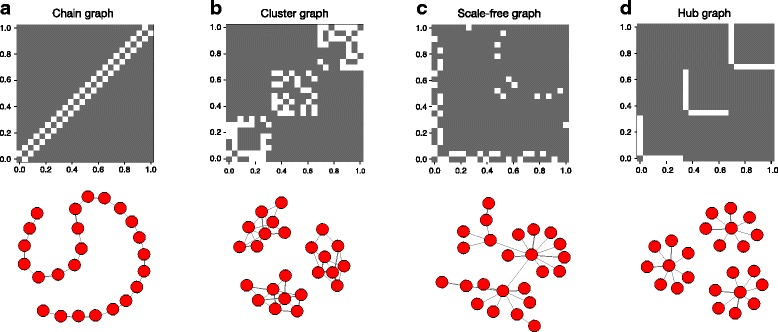
Illustration of the four different graphs that have been used in our study. Shown are the adjacency matrices of the graphs and their corresponding graph topologies. **a** Chain graph with maximum degree of 2. **b** Cluster graph which consists of three disjoint subgraphs. **c** Scale-free graph (Barabasi-Albert graph). **d** Hub graph, also known as a star graph

In this section, we compare the correlation- and partial correlation-based methods on different graph topologies based on synthetic data. For this purpose, we have generated the synthetic data and a workflow of data generation is illustrated in Fig. 4. In the following, we shortly describe several graphs used in the comparison which are illustrated in Fig. 5:

All graphs used in the comparison have the same dimension *p* and are generated from the adjacency matrices with the size *p*×*p*.

1. *Chain graph*. The graph corresponds to a tridiagonal adjacency matrix where each row and column consist of one or two non-zero entries which correspond to the graph with the maximum degree of 2. The graph consists of *p*−1 number of edges.
2. *Cluster graph*. The rows/columns of the adjacency matrix are evenly partitioned into *l* disjoint submatrices. Here, we denote them as *U*_*i*_,*i*=1,…,*l*. Since they are disjoint, we can write *U*_1_∪*U*_2_∪,…,∪*U*_*l*_={1,…,*p*} and the corresponding graph contains *p*(*p*/*l*−1)*P*/2 number of edges, where *P* is the probability of the edge between any two nodes in a subgraph. If probability *P*=1, then disjoint subgraphs are fully connected. Decreasing *P* allows to generate sparse subgraphs.
3. *Scale-free graph* (Barabasi-Albert model) ([26, 27]). The degree of the graph follows a power law distribution (30). The graph generation is based on a preferential attachment and starts with *m*_0_ nodes. The new nodes with *m*≤*m*_0_ edges are added to *m*_0_ existing nodes in the graph. A new node is added to the existing node *i* depending on the degree *k*_*i*_ with the probability $P(k_{i}) = k_{i}/\sum _{j}^{}k_{j}$. The graph contains *p*−1 edges.
4. *Hub graph*. The rows/columns of the adjacency matrix are evenly partitioned into *l* disjoint groups as in the cluster graph, *U*_1_∪*U*_2_∪,…,∪*U*_*l*_={1,…,*p*}. At each disjoint subgraph, a hub node has more connections to other nodes, whereas the other nodes have only one connection. Since a partitioning is even, every subgraph contains the same number of nodes and edges.

All graphs are generated using R package *huge* [32].

### Comparison of methods based on optimal predictions

**Fig. 6:**
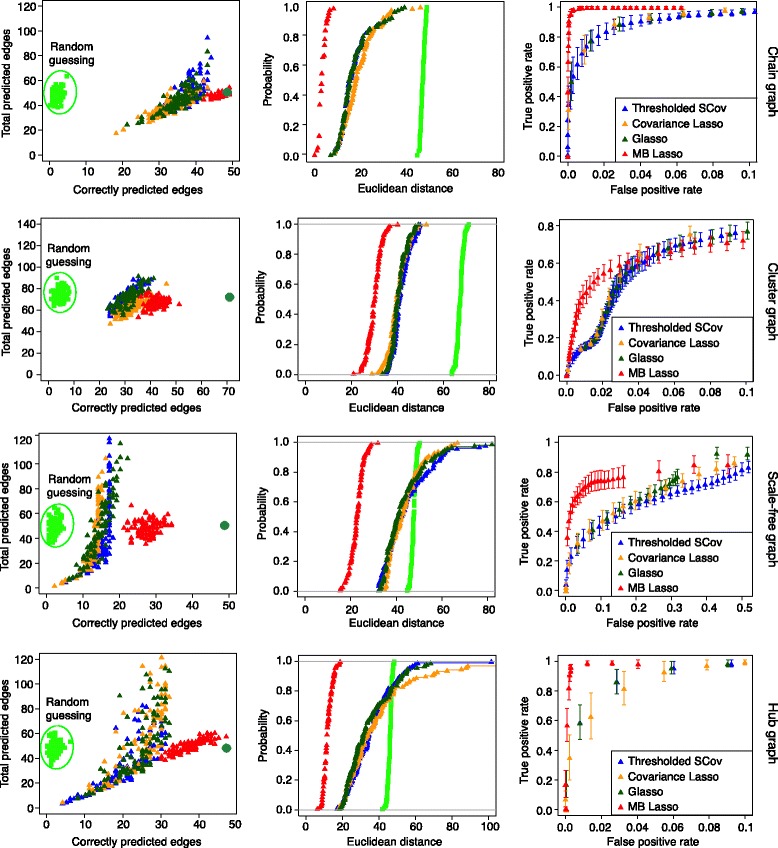
Predictions by the nodewise regression Lasso (MB Lasso), the graphical Lasso (Glasso), the covariance Lasso, the thresholded sample covariance matrix (Thresholded SCov), and the random guessing using the synthetic data generated from four graph types (chain, cluster, scale-free, and hub graphs). Illustrated are predicted edges (resampled 100 times) and true edges (*dark green circle*) on correctly predicted vs total predicted axes (*left*). The Euclidean distances from true edges to predicted edges are summarized in terms of cumulative distribution which indicates the probability of the Euclidean distances (*middle*). Performances of methods are also assessed using traditional ROC curves (resampled 20 times)

First, we performed the comparison on an ideal case where the underlying graph is known and one can optimize predictions based on the given graph (Fig. 6). This way, one can judge the performance of methods under optimal conditions. Since the adaptive Lasso is an adaptive version of nodewise regression method, it is not considered for comparison in this setting.

For all four graphs, we choose the graph size *p*=50 and generate the dataset with the sample size *n*=30. To account uncertainty in the data generation, we resample the data 100 times and perform the graph reconstruction with 100 datasets each of size *p*=50. This allows us to assess the performance of methods in the presence of noise. For better illustration purposes, we plot predicted edges on the correctly predicted vs total predicted axis (Fig. 6 (left)). In addition to methods, we perform predictions by random guessing, which is used for a quality control in our study. To assess the quality of predictions produced by different methods, we compute Euclidean distances from individual edge predictions to true edges as

42 $$ d_{E} = \sqrt{(T_{R} - C_{\text{pred}})^{2} + (T_{R} - T_{\text{pred}})^{2}},  $$

where *T*_*R*_ denotes true edges in the true graph, *C*_pred_ and *T*_pred_ represent correctly predicted and total predicted edges, respectively. We then compute the cumulative distribution of *d*_*E*_ (Fig. 6 (middle)).

To further compare four methods, we also compute the receiver operating characteristics (ROC)

$$\text{TPR}=\frac{\text{TP}}{\mathrm{TP+FN}}, \hspace*{1cm} \text{FPR} = \frac{\text{FP}}{\mathrm{FP+TN}}, $$

where TPR is a true positive rate defined as a ratio of predicted true positives TP to total positives TP+FN. False positive rate FPR is the ratio of predicted false positives FP to total false positives FP+TN. The nodewise regression Lasso performs well on the chain graph with *E*=49 edges which is regarded as simplest (Fig. 6 (first top panel)). Other methods predict about 35 to 40 edges correctly, whereas the nodewise regression Lasso produces almost perfect predictions. On the scale-free graph, the nodewise regression Lasso performs best among four methods. The prediction accuracy is about more than half of true edges for the nodewise regression Lasso and less than half for three remaining methods. The three methods predict almost a similar number of edges out of which 10 to 20 are correct edges. From ROC curves, one can see that initially all three methods perform similarly, but later, the graphical Lasso starts outperforming the thresholded sample covariance and the covariance Lasso. Since the scale-free graph contains more highly connected nodes (maximum degree *k*_max_ = 13) compared to other graphs, the prediction accuracy of all methods reduces in comparison to chain and cluster graphs thereby being close to predictions by random guessing. For the cluster graph, we set the probability of the edge between any two nodes to *P*=0.3, so that the resulting graph contains less hub nodes as possible (*k*_max_=4). The nodewise regression Lasso predicts on average 40 true edges out of 70, whereas other methods predict 30. In case of the hub graph, where we have 10 disjoint subgraphs with 10 hub nodes, the predictions of the nodewise regression Lasso are again best among other methods by predicting about 40 true edges out of 50. In contrast, the remaining three methods only predict a half of all true edges. We observe that the thresholded covariance, the covariance Lasso, and the graphical Lasso predict almost a similar number of true edges in all four graphs. In contrast, the nodewise regression Lasso performs best compared to other methods in all four graphs. Our comparison metrics are based on the control of false positive edges, and a similar observation was published earlier in the work of Peng et al. [33], where the authors showed that the nodewise regression Lasso performs better than the graphical Lasso when controlling for false discovery rate.

## Comparison of methods when underlying graph is not known

In this section, we are going to discuss how the methods perform when the underlying graph is not given. This is a typical case in applications where the underlying graph is not known, and a challenge is to infer the graph based on the data. We are therefore going to discuss available methods that allow the selection of the optimal threshold for the sample covariance matrix and optimal regularizations for covariance Lasso and adaptive Lasso methods. Because, a cross-validated choice of the penalty parameter in nodewise regression and graphical Lasso methods leads to overestimation problem, we consider selecting the penalty from the adaptive Lasso by cross-validation which gives a sparser solutions compared to former methods. We already introduced these methods in previous sections and are going to discuss how they perform in practice. For comparison, we choose the same settings: *p*=50 and *n*=30.

### Scale-free criteria-based thresholding of sample covariance matrix

**Fig. 7:**
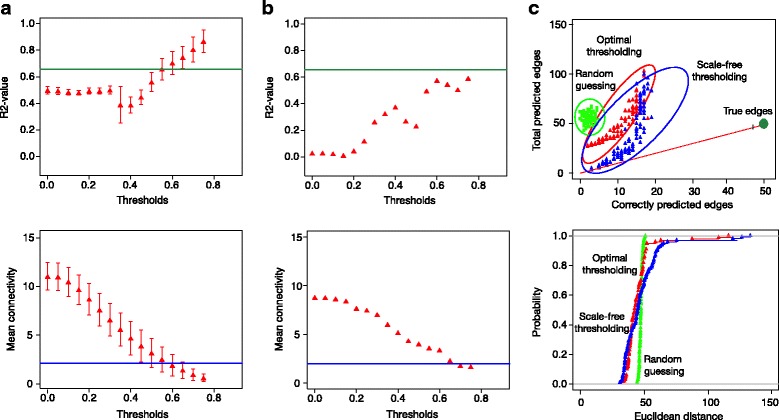
Selecting the optimal threshold value based on *R* ^2^ and mean degree values when the underlying graph is scale-free. **a** Hard-thresholding on the sample covariance matrix, ***S*** computed from data. **b** Hard-thresholding on the covariance matrix obtained from the true graph. *Green* and *blue lines* indicate *R* ^2^ values and mean degree values from the true graph, respectively. **c** Predictions with hard-thresholding of the sample covariance matrix

In this section, we discuss the application of scale-free thresholding in comparison to the optimal thresholding which is based on the true graph. We compute *R* ^2^ values and mean degree values $\bar {k}$ for various thresholds uniformly selected from [0,1]. For a reference graph, we also compute the *R* ^2^ value (green line) and the mean degree value $\bar {k}$ (blue line) of the true graph. As illustrated in Fig. 7 a, higher *R* ^2^ values are achieved for the threshold higher than 0.5 which can be compared to that of the true graph (green line). The corresponding mean degree value for the threshold higher than 0.5 is also close to that of the true graph (blue line). To compare how well the threshold is selected, we further perform hard-thresholding on the true covariance matrix and compute *R* ^2^ and mean degree values (Fig. 7 b). Since the graph for the true covariance matrix is fully connected, without thresholding, it returns low *R* ^2^ and high mean degree values. High *R* ^2^ values are achieved for the threshold higher than 0.5 as it was observed in the scale-free selection case (Fig. 7 a). In particular, the mean degree values close to true mean values are also attained approximately at the same threshold. In practical applications, when inferring a gene co-expression graph from microarray data, it is usually suggested to select the threshold with high *R* ^2^ values and low mean degree values. In particular, for a high-dimensional case with thousand genes, these two metrics show saturation for high *R* ^2^ and low mean degree values. Although in our case there is no saturation effect, it is possible to select the threshold to be 0.6, for which the *R* ^2^ value is high and the mean degree value is low. Furthermore, we perform simulations with this threshold and compute the number of true edges in the thresholded graph (Fig. 7 c). As the plot indicates, the selected threshold is nearly optimal giving predictions close to optimal ones. Despite it gives results close to the optimal ones, best threshold predictions are almost as bad as the results of random guessing. It is noteworthy that, in our simulations, this method was shown to work well when the sample size is larger than the variable size (*p*<*n*). Since we only consider the *p*>*n* case in our study, the results are not shown.

Theoretically, high *R* ^2^ values can be achieved only for scale-free graphs and not applicable for other graph types. We also show that it is not possible to attain high *R* ^2^ values with other graph types used in our study (results are not shown here).

### Cross-validation with covariance Lasso

**Fig. 8:**
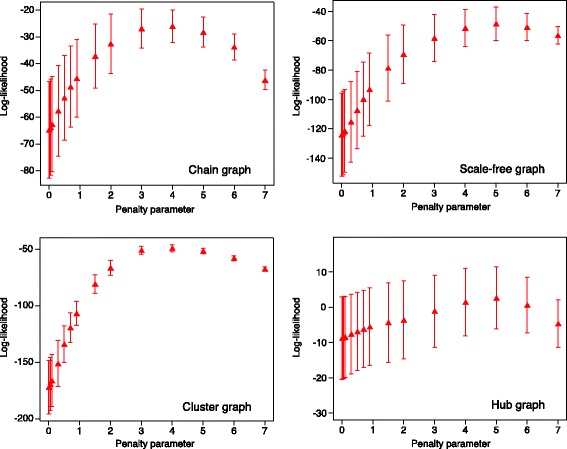
Selecting penalty parameters in the covariance Lasso by cross-validation approach for four graph types. The log-likelihood values are computed for a range of penalty parameters. Cross-validation selects the penalty parameter for which the log-likelihood attains a maximum value

To choose the penalty parameter *λ*_cov_ from the data, we compute it by cross-validation procedure. We perform fivefold cross-validation and select the penalty parameter that maximizes the log-likelihood function in (31). Figure 8 depicts computed likelihood values with the penalty parameters selected from a range *λ*_cov_∈[0,7]. The results show that the maximum likelihood values for all graphs exist almost in a close range of the penalty parameter. For chain and cluster graphs, the maxima are attained between *λ*_cov_=3 and *λ*_cov_=5, whereas for scale-free and hub graphs, between *λ*_cov_=4 and *λ*_cov_=6. Therefore, the penalty parameters for further simulations, we have chosen from these ranges where the maximum for the log-likelihood is attained. We then performed the covariance graph estimation using these penalty parameters. Unfortunately, we observe that in all cases, these penalty values lead to the overestimation of the graph. In particular, a lot of false positive edges are selected in the estimated graph.

### Cross-validation with adaptive Lasso

**Fig. 9:**
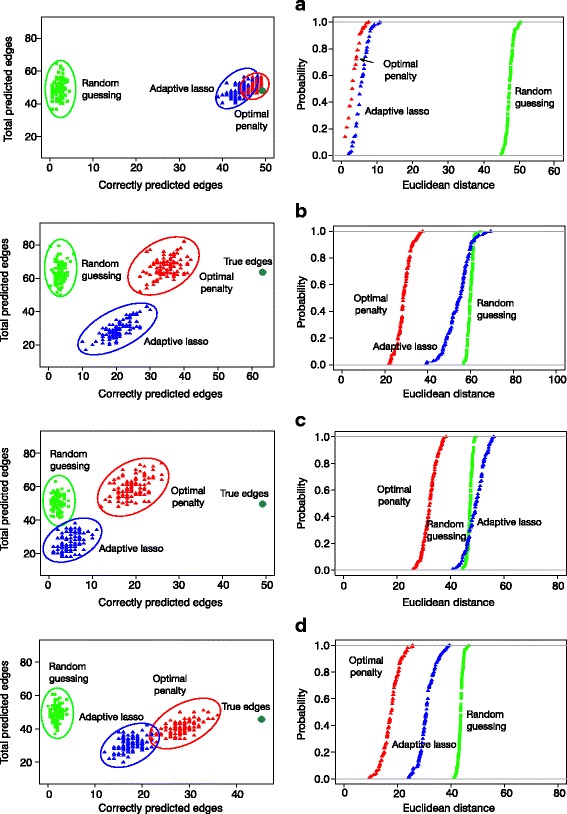
Predictions based on the adaptive Lasso with the penalty parameter chosen via cross-validation, the nodewise-regression with the optimal penalty, and the random guessing. Depicted are predictions for **a** chain graph, **b** cluster graph, **c** scale-free graph, and **d** hub graph

In order to select a suitable penalty value, we perform cross-validation with the adaptive Lasso (41). We observe that cross-validation with the adaptive Lasso performs very well on chain graphs (Fig. 9 a), where the predictions (blue) are in a close range to optimal predictions (red). For cluster and hub graphs, the method performs poorly compared to the optimal one, but still returns better results in contrast to random guessing (Fig. 9 b, d). However, in the scale-free graph, the method performs poorly giving predictions almost in the same range as random guessing (Fig. 9 c). But one can observe from the scatter plot that on average, the method gives slightly more true positives but at the same time predicts less false positive edges compared to random guessing. One also has to be aware that the scale-free graph used in our study contains far more hub nodes which have more connected edges compared to other nodes. This type of graphs is very difficult to infer under the setting *p*>*n*. Other graphs used in the study contain less number of hub nodes and the method performs well on these graphs. For example, the maximum degree of the chain graph is *k*_max_=2, for the cluster graph *k*_max_=4, for the hub graph *k*_max_=9, and for the scale-free graph *k*_max_=13. Therefore, we observe that the penalty selection under cross-validation with the adaptive Lasso is highly dependent on the number of hub nodes in the graph. We also have to mention that the adaptive Lasso method does not take any prior information about the graph topology and applies the uniform penalty on all edges in the graph, which is also a major drawback of the method when applied to graphs which contain more hub nodes. This observation was also reported earlier in the other studies [34–36].

## Effect of correlation strength on the performance of methods

In this section, we are going to discuss the role of correlation strength on the performance of methods. It has been shown that a magnitude of correlations should be bounded from below in order for the method to give consistent predictions [13]. It is known that if data variability is less, then large sample size is required to increase an estimation accuracy. If the sample size is limited, which is often the case in biomedical applications, then it is possible to increase the prediction accuracy by increasing the variability in the data so that correlation information between variables is high. In this section, we examine how prediction accuracy of methods is affected with changes in data variability. For this purpose, we generate several datasets from the correlation matrices with different correlation magnitudes and then perform the graph reconstruction with four methods on these datasets. To generate datasets with a different degree of correlation, we use the method introduced in [32].

Let ***A*** be the *p*×*p* adjacency matrix which consists of binary values and represents a certain graph. To induce different correlation strengths in the data, we first multiply ***A*** with some scalar *w*>0 and convert the resulting matrix into the positive definite matrix

43 $$ \boldsymbol{\hat{\!A}} = w\boldsymbol{A} + \gamma \boldsymbol{I},  $$

where *γ*=| min(*λ*_*i*_)|+*ε*,*i*=1,…,*p* and *ε*>0. Here *λ*_*i*_ are the eigenvalues of the matrix *w* ***A***. Then, we compute the correlation matrix by

44 $$ \boldsymbol{C} = \boldsymbol{\Lambda}^{-\frac{1}{2}}\,\boldsymbol{\hat{\!A}}^{-1}\boldsymbol{\Lambda}^{-\frac{1}{2}} = \boldsymbol{\Lambda}^{-\frac{1}{2}}(w\boldsymbol{A} + \gamma \boldsymbol{I})^{-1}\boldsymbol{\Lambda}^{-\frac{1}{2}},  $$

where ***Λ*** is the matrix of diagonal elements of the covariance matrix $\,\boldsymbol {\hat {\!A}}^{-1}$. As a measure of the correlation magnitude, we define $\sigma =(\sqrt {\smash [b]{\text {var}(C_{ij}))}}, \ i, j = 1,\ldots,p$. Here, the different values of *w* allow to generate the correlation matrices with different magnitudes. The correlation matrix is then used to generate datasets using the procedure described in Fig. 4.

**Fig. 10:**
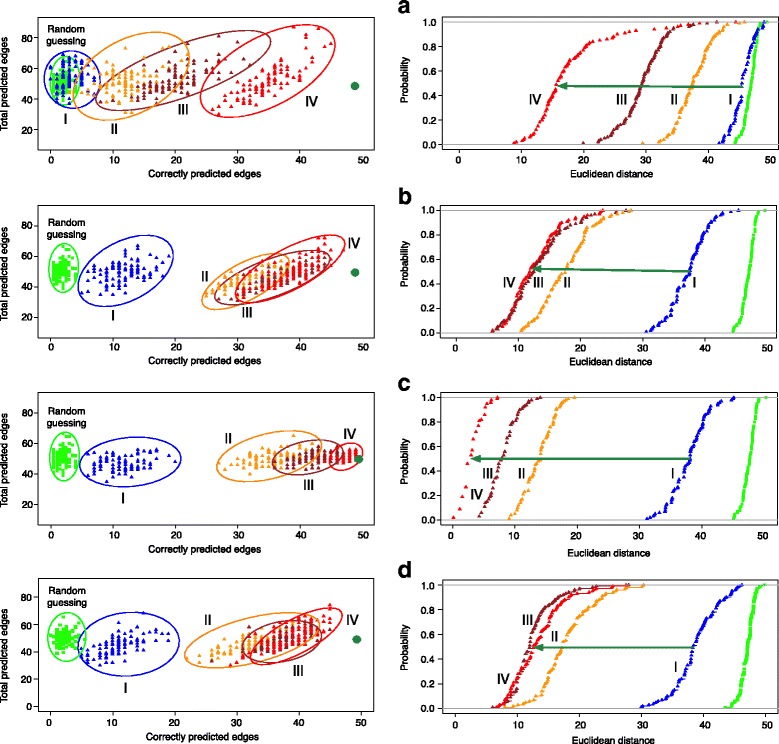
Influence of correlation strength on predictions in case of the chain graph (*p*=50, *n*=30). **a** Thresholded sample covariance matrix. **b** Covariance Lasso. **c** Nodewise regression Lasso. **d** Graphical Lasso Illustrated are predictions with the different correlation strength as indicated with (*I*) low correlation, *σ*≈0.15 (*II*) moderate correlation, *σ*≈0.19 (*III*) moderate-high correlation, *σ*≈0.22 and (*IV*) high correlation, *σ*≈0.36

Figure 10 depicts optimal predictions produced by four methods in case of different correlation strengths on the chain graph. Sensitivity of predictions by four methods computed as the average ratio of correctly predicted to total predicted edges is given in Table 2. In this case, we choose the optimal threshold and the penalty based on the shortest Euclidean distance from true edges. When the magnitude of correlations is low (standard deviation, *σ*≈0.15, colored in blue), the performance of methods is relatively poor. In this regime, all methods predict about 1/4 of correct edges. Increasing the magnitude of correlation positively affects the performance of all methods (II, III, and IV). For instance, at *σ*≈0.19, the sensitivity of the thresholded sample covariance matrix predictions increases from 0.23 to 0.67. In this regime, the sensitivity of the covariance Lasso increases from 0.24 to 0.72 (12 to 30 edges), while the sensitivity for the nodewise regression Lasso and the graphical Lasso increases from 0.24 to 0.7 (from 13 to 35 edges). The accuracy of covariance Lasso predictions does not change so much from II to IV, indicating a saturation effect of the method. The saturation effect is also observed for the thresholded sample covariance matrix from (III) to (IV). In contrast, the sensitivity of the nodewise regression Lasso and the graphical Lasso predictions increases with the increasing correlation strength. In the regime (III), the sensitivity of the nodewise regression Lasso is about 0.83, whereas at (IV), it is almost 0.93. The sensitivity of the graphical Lasso increases from 0.75 (III) to 0.82 (IV).

**Table 2:** Sensitivity of predictions computed by four methods calculated as the average ratio of correctly predicted to total predicted edges

Correlation strength	*σ*≈ 0.15 (I)	0.19 (II)	0.22 (III)	0.36 (IV)
Thresholded sample covariance	0.23	0.67	0.73	0.73
Covariance Lasso	0.24	0.72	0.8	0.77
Nodewise regression Lasso	0.24	0.7	0.83	0.93
Graphical Lasso	0.25	0.7	0.75	0.82

## Conclusions

High-dimensional graph reconstruction methods have attracted much scientific interest over the last years and continue to be investigated further. In this work, we analyze the relation between concentration and covariance graphs and further conduct the detailed comparison between various graph reconstruction methods designed to infer concentration as well as covariance graphs. Our analytical study shows that it is possible to establish a link between these two graphs using Neumann series. In particular, we show the entry-wise relation between the entries of the covariance matrix and the transitive closure matrix associated to the concentration graph. We analytically demonstrate this relation for a star graph. Moreover, we analytically demonstrate a graph property that the covariance graph associated to the correlation matrix can be shown as the minimum transitive closure of the concentration graph. We also show a small scale demonstration for a three-node graph. Eventually, this property can be exploited to infer edge weights of the covariance graph directly from edge weights of the concentration graph. Currently, it has been shown for a star graph, but can be extended to other graph types too.

Furthermore, we performed the analytical and numerical studies on recently published network deconvolution and network silencing methods [10, 11]. In particular, we derived the analytical solution to the network deconvolution problem by exploiting facts from Kac-Murdock-Szëgo matrix. We also give more insights about the role of the scaling parameter which has been studied only numerically in the original study. Moreover, we conducted a detailed comparison of the methods designed to reconstruct covariance and concentration graphs on different graph topologies. In order to resemble the high-throughput experiments, we designed our simulation experiments with more variables than samples (*p*>*n*). We showed that the nodewise regression Lasso allows to select a consistent penalization which controls the number of false positives compared to the thresholded sample covariance, the covariance Lasso methods, and the graphical Lasso. The adaptive version of nodewise regression Lasso also allows to control the rate of false positives better than correlation-based methods when the penalty parameter is chosen via cross-validation.

## Additional file

Additional file 1 Supplementary material for “Graph reconstruction using covariance based methods”. (PDF 50 kb)
