# Supplementary material for: Graph reconstruction using covariance-based methods
Source: EURASIP J Bioinform Syst Biol. 2016 Nov 23;2016:19. doi: 10.1186/s13637-016-0052-y (PMC5121191; doi:10.1186/s13637-016-0052-y)
Supplement: Supplementary file 1 — Supplementary material for “Graph reconstruction using covariance based methods”. (PDF 50 kb) [file 13637_2016_52_MOESM1_ESM.pdf]

Additional file 1

Proof of Proposition 1

We consider a star graph with p nodes and hence $p-1$ edges. We denote the hub node of the graph by k. In order to reach any node $j \neq k$ from any node $i \neq k$, one needs to pass through k and hence takes two hops. Through symmetry we only need to consider the following four cases: (i) $i \to k \to j$, (ii) $i \to k$, (iii) $i \to i$ and (iv) $k \to k$. Due to the similiarity of the steps, we only detail the steps for case (i). Generally, for any (i, j) the transitive closure (8) (of the main text) in component form without exploiting the graph structure reads

$$A^*_{ij} = A_{ij} + \sum_{s=1}^{\infty} \left(\sum_{m_1=1}^{p} \cdots \sum_{m_{s-1}=1}^{p} A_{im_1} A_{m_1 m_2} \ldots A_{m_{s-1} j} \right). \tag{1}$$

For case (i) the lowest order contribution to A^*_{ij} is quadratic and corresponds to the path of minimal length connecting i and j. For a star graph that path is unique and hence any non-zero higher order term in (1) must contain all edges of that path at least once. Hence, one can factor out those edges and can conclude that the remaining edges need to form cycles. Finally, observing that a star graph only contains trivial cycles and that after appropriate edge permutation[1] they are of the form $k \to l \to k$ we can write

$$\begin{aligned} A^*_{ij} &= A_{ik} A_{kj} \left[1 + \sum_{s=1}^{\infty} \sum_{m_1=1}^{p} \cdots \sum_{m_{s-1}=1}^{p} A_{km_1} A_{m_1 k} \ldots A_{km_{s-1}} A_{m_{s-1} k} \right] \\ &\equiv A_{ik} A_{kj} (1 + Z_{kk}), \end{aligned} \tag{2}$$

where we introduce the cycle contribution Z_{kk}. Hence, any path contributing to A^*_{ij} can be seen as a minimal path decorated with trivial cycles, represented by Z_{kk}. Every summand in Z_{kk} can now be characterized by counting how many times the $p-1$ available cycle terms $M_l \equiv A_{kl} A_{lk}$ are used. Hence, for a cycle budget n determined by the power s in $\boldsymbol{A}^s$ (i.e. for case (i) the cycle edge count in Z_{kk} is the even number $s-2$ and hence the number of cycles is $n = (s-2)/2$), one is left with counting how many ways we can distribute n cycles over the

[1] We note that the path weight computation is commutative in that two distinct paths showing the same edge utilization (which edge was used how many times) will receive the same weight; that is, any edge permutation keeps the weight invariant.

$p-1$ different cycle types M_l. That is,

$$\begin{aligned} A^*_{ij} &= A_{ik}A_{kj}\sum_{n=0}^{\infty}\sum_{l_1=0}^{n}\cdots\sum_{\substack{l_{p-1}=0\\ l_1+\cdots+l_{p-1}=n}}^{n}\binom{n}{l_1,l_2,\ldots,l_{p-1}}M_1^{l_1}M_2^{l_2}\ldots M_{p-1}^{l_{p-1}} \\ &= A_{ik}A_{kj}\sum_{n=0}^{\infty}(M_1+\cdots+M_{p-1})^n = \frac{A_{ik}A_{kj}}{1-\sum_{l=1}^{p}M_l} \\ &= \frac{A_{ik}A_{kj}}{1-\sum_{l=1}^{p}A_{kl}A_{lk}} = A_{ik}A_{kj}/c \quad \text{with} \quad c\equiv 1-\sum_{l=1}^{p}A_{kl}A_{lk}, \end{aligned} \tag{3}$$

where we assumed convergence of the geometric series through $|M_1+\cdots+M_{p-1}|<1$. Using the same procedure we can write the closed form expressions for the remaining cases:

$$\begin{aligned} (ii) &\quad i\to k: \ \tilde{A}^*_{ij} = A_{ik}/c, \\ (iii) &\quad i\to i: \ \tilde{A}^*_{ii} = A_{ik}A_{ki}/c, \\ (iv) &\quad k\to k: \ \tilde{A}^*_{kk} = c^{-1}\sum_{l=1}^{p}A_{kl}A_{lk} = (1-c)/c = 1/c-1. \end{aligned}$$

□

Proof of Proposition 2

Proof. With Proposition 1 and the construction for the covariance matrix $\mathbf{\Sigma} = \boldsymbol{D}^{-1}(\boldsymbol{I}+\boldsymbol{A}^*)\boldsymbol{D}^{-1}$ with $\boldsymbol{D}$ the diagonal scaling matrix entries $(d_1,\ldots,d_p)$, we obtain for the four cases of the star graph

$$\begin{aligned} \Sigma_{ij} &= \frac{A_{ik}A_{kj}}{cd_id_j},\ \Sigma_{ik} = \frac{A_{ik}}{cd_id_k},\ \Sigma_{kj} = \frac{A_{kj}}{cd_kd_j}, \\ \Sigma_{ii} &= (1+A_{ik}A_{ki}/c)\frac{1}{d_i^2} = (1-\sum_{\substack{l=1\\ l\neq i}}^{p}A_{kl}A_{lk})\frac{1}{cd_i^2} = \frac{c_i}{cd_i^2}, \\ \Sigma_{kk} &= (1+1/c-1)\frac{1}{d_k^2} = \frac{1}{cd_k^2}, \end{aligned} \tag{4}$$

for $i,j\in\{1,\ldots,p\}\setminus\{k\}$ with $c_i = 1-\sum_{l=1,l\neq i}^{p}A_{kl}A_{lk}$. Now we perform an element-wise conversion of entries of the covariance matrix $\mathbf{\Sigma}$ to entries of the correlation matrix $\boldsymbol{C} = \mathbf{\Lambda}^{-1}\mathbf{\Sigma}\mathbf{\Lambda}^{-1}$ with $\mathbf{\Lambda}$ a diagonal scaling matrix with entries

$$\Lambda_{ii} = \sqrt{\frac{c_i}{cd_i^2}},\ \Lambda_{kk} = \sqrt{\frac{1}{cd_k^2}},\ i\in\{1,\ldots,p\}\setminus\{k\} \tag{5}$$

and compute the normalized entries

$$
\begin{aligned}
C_{ij} &= \Lambda_{ii}^{-1}\Sigma_{ik}\Lambda_{jj}^{-1} = \sqrt{\frac{cd_i^2}{c_i}}\frac{A_{ik}A_{kj}}{cd_id_j}\sqrt{\frac{cd_j^2}{c_j}} = \frac{A_{ik}A_{kj}}{\sqrt{c_ic_j}} \\
C_{ik} &= \Lambda_{ii}^{-1}\Sigma_{ij}\Lambda_{kk}^{-1} = \sqrt{\frac{cd_i^2}{c_i}}\frac{A_{ik}}{cd_id_k}\sqrt{d_k^2c} = \frac{A_{ik}}{\sqrt{c_i}} \\
C_{kj} &= \Lambda_{kk}^{-1}\Sigma_{kj}\Lambda_{jj}^{-1} = \sqrt{d_k^2c}\frac{A_{kj}}{cd_kd_j}\sqrt{\frac{cd_j^2}{c_j}} = \frac{A_{ik}}{\sqrt{c_j}}.
\end{aligned}
\tag{6}
$$

Now define a graph $\hat{G} = (\Gamma, E, \hat{w})$ which contains the same set edges as a star graph G with $\hat{w}_{ik} = C_{ik}$ and $\hat{w}_{kj} = C_{kj}$. Then the edge weights of a minimal transitive closure of the graph $T(\hat{G})$ are defined as

$$\tilde{w}_{ik} = \hat{w}_{ik},\ \tilde{w}_{kj} = \hat{w}_{kj} \text{ and } \tilde{w}_{ij} = \hat{w}_{ik}\hat{w}_{kj}$$

from which it directly follows

$$\tilde{w}_{ik} = C_{ik},\ \tilde{w}_{kj} = C_{kj} \text{ and } \tilde{w}_{ij} = C_{ik}C_{kj},$$

hence $T(\hat{G}) = G'$. □

Determining the concentration graph by hard-thresholding the covariance matrix

Here, we ask if it is possible to threshold the covariance graph and obtain a similar graph as in the concentration graph. For a demonstration, we consider a chain graph and conduct simulation experiments on the correlation matrix rather than the covariance matrix.

In particular, we analyze two cases:
(i) The thresholded covariance graph is a good approximation of the concentration graph.
(ii) The thresholded covariance graph is not a good approximation of the concentration graph.

In order to examine these two points, we consider a simple four-node chain graph (Figure 1d (left)).

Case (i): In this case, we expect that in the covariance graph, the indirect edge weights are smaller than the direct edge weights. Because when performing a thresholding operation, the indirect edges will be eliminated first and therefore it is possible to obtain a good thresholded graph similar to the concentration graph. The relationship between concentration and covariance graphs we established in the previous section allows to infer the thresholding scenario directly from the edge weights of the concentration graph. We therefore check whether

the following conditions hold:

$$\begin{aligned}
A_{12}A_{24} < A_{12} &\Rightarrow C_{12}C_{24} < C_{12}\\
A_{12}A_{24} < A_{24} &\Rightarrow C_{12}C_{24} < C_{24}\\
A_{12}A_{24} < A_{13} &\Rightarrow C_{12}C_{24} < C_{13}\\
A_{12}A_{13} < A_{12} &\Rightarrow C_{12}C_{13} < C_{12}\\
A_{12}A_{13} < A_{24} &\Rightarrow C_{12}C_{13} < C_{24}\\
A_{12}A_{13} < A_{13} &\Rightarrow C_{12}C_{13} < C_{13}\\
A_{12}A_{13}A_{24} < A_{12} &\Rightarrow C_{12}C_{13}C_{24} < C_{12}\\
A_{12}A_{13}A_{24} < A_{24} &\Rightarrow C_{12}C_{13}C_{24} < C_{24}\\
A_{12}A_{13}A_{24} < A_{13} &\Rightarrow C_{12}C_{13}C_{24} < C_{13}
\end{aligned} \tag{7}$$

The condition (7) indicates that in the concentration graph, if the absolute values of indirect edge weights are smaller than those of direct edges, then this condition also holds for the covariance graph. If this condition holds, then by thresholding one will get edges in the covariance graph corresponding to direct edges in the concentration graph. To check this condition, we fix the edge values in the concentration matrix corresponding to the four-node chain graph: $A_{12} = A_{13} = 0.4$ and $A_{24} = 0.3$ so that $||\boldsymbol{A}|| < 1$ and (7) hold,

$$\boldsymbol{A} = \begin{pmatrix} 0 & 0.4 & 0.4 & 0 \\ 0.4 & 0 & 0 & 0.3 \\ 0.4 & 0 & 0 & 0 \\ 0 & 0.3 & 0 & 0 \end{pmatrix}$$

We then compute the covariance matrix using $\boldsymbol{\Sigma} = \boldsymbol{D}^{-1}(\boldsymbol{I} + \boldsymbol{A}^*)\boldsymbol{D}^{-1}$ with $\boldsymbol{A}^* = \sum_{m=1}^{\infty} \boldsymbol{A}^m = \boldsymbol{A}(\boldsymbol{I} - \boldsymbol{A})^{-1}$, and further convert it to the correlation matrix

$$\boldsymbol{C} = \begin{pmatrix} 1 & 0.46 & 0.44 & 0.15 \\ 0.46 & 1 & 0.20 & 0.33 \\ 0.44 & 0.20 & 1 & 0.07 \\ 0.15 & 0.33 & 0.07 & 1 \end{pmatrix}.$$

Hence, we observe $C_{12} = 0.46$, $C_{13} = 0.44$ and $C_{24} = 0.33$, which satisfies (7). This implies that it is possible to find an optimal threshold that separates direct edges from indirect ones. In order to get the same sparsity as in the concentration graph, we set all the values smaller than $C_{ij} < 0.3$ $(i, j = 1, 2, 3, 4)$ and the diagonal elements to zero and get a thresholded correlation matrix

$$\boldsymbol{C}_T = \begin{pmatrix} 0 & 0.46 & 0.44 & 0 \\ 0.46 & 0 & 0 & 0.33 \\ 0.44 & 0 & 0 & 0 \\ 0 & 0.33 & 0 & 0 \end{pmatrix}$$

We see that the thresholded correlation matrix has the same zero patterns as in the concentration matrix. Analogously, the corresponding covariance graph

contains the same edges as in the concentration graph (Figure 1e (left)). We can conclude that when the condition (7) for this example holds, it is possible to obtain a good approximation of the concentration graph by the covariance graph.

Case (ii): Next, we check the case when the covariance graph is not a good estimator for the concentration graph. We verify the case when one sub-condition of (7) is violated. In this case, we choose $A_{12} = A_{24} = 0.6$ and $A_{13} = 0.2$, so that $A_{12}A_{24} > A_{13}$. Performing the same procedure as before, we obtain the correlation matrix

$$\boldsymbol{C} = \begin{pmatrix} 1 & 0.77 & 0.3 & 0.58 \\ 0.77 & 1 & 0.23 & 0.76 \\ 0.3 & 0.23 & 1 & 0.18 \\ 0.58 & 0.76 & 0.18 & 1 \end{pmatrix}$$

where $C_{12} = 0.77$, $C_{24} = 0.76$ and $C_{13} = 0.3$, from which we get $C_{12}C_{24} > C_{13}$. This indicates that the condition (7) is violated

$$A_{12}A_{24} > A_{13} \Rightarrow C_{12}C_{24} > C_{13} \tag{8}$$

To get the same sparsity as in the concentration matrix, we choose the threshold $C_{ij} < 0.4$ $(i, j = 1, 2, 3, 4)$ and and compute the thresholded correlation matrix

$$\boldsymbol{C}_T = \begin{pmatrix} 0 & 0.77 & 0 & 0.58 \\ 0.77 & 0 & 0 & 0.76 \\ 0 & 0 & 0 & 0 \\ 0.58 & 0.76 & 0 & 0 \end{pmatrix}$$

So, the predicted edge weights are $C_{12} = 0.77$, $C_{24} = 0.76, C_{13} = 0$ and $C_{14} = 0.58$. Thresholding predicts two direct edges correctly C_{12} and C_{24}, fails to predict C_{13} and additionally predicts a false positive edge C_{14} which is not present in the concentration graph. In this case, the thresholded correlation matrix is not a good estimator for graph topology (Figure 1e (middle and right)).

Next, we extended the comparison to high-dimensional chain graphs. In this case, the condition (7) can be extended to p number of variables:

$$\begin{gathered} A_{i(i+1)}A_{(i+1)(i+2)} < A_{i(i+1)} \Rightarrow C_{i(i+1)}C_{(i+1)(i+2)} < C_{i(i+1)}, \\ A_{i(i+1)}A_{(i+1)(i+2)} < A_{(i+1)(i+2)} \Rightarrow C_{i(i+1)}C_{(i+1)(i+2)} < C_{(i+1)(i+2)}, \\ \vdots \\ A_{12}A_{23} \cdots A_{(p-1)p} < A_{k(k+1)} \Rightarrow C_{12}C_{23} \cdots C_{(p-1)p} < C_{k(k+1)}, \\ i = 1, \ldots, p-2,\ k = 1, \ldots, p-1. \end{gathered} \tag{9}$$

In order to select edges that satisfy these conditions, we choose a chain graph with $p = 500$, $A_{i(i+1)} \sim \mathcal{N}(\mu, \sigma^2)$, $i = 1, \ldots, p-1$ and assume $\mu = 0.4$ and $\sigma = 0.0005$. This way selected edge weights are scattered around the mean

$\mu = 0.4$ and satisfy (9). Using the same operations, we computed distributions of direct and indirect edges in the covariance graph (Figure 1f and g). It can be observed that when $A_{i(i+1)}$ are sampled from the narrow distribution with $\sigma = 0.0005$, the distributions of direct and indirect edges in the covariance graph are narrow and clearly distinguishable (Figure 1f). In this case, it is possible to select a good threshold that can separate these two distributions.

Next, we consider the case with $\mu = 0.4$ and $\sigma = 0.5$, where the edge weights $A_{i(i+1)}$ are sampled from the broad distribution. In this case, both the direct and indirect edges are highly overlapping and it is hard to separate two distributions with the optimally selected threshold (Figure 1g). One can find many edges that violate the condition (9). Consequently, the thresholded covariance graph contains some false positive edges and lacks some true edges after hard-thresholding. Eventually, the conditions (9) also hold for star graphs which we numerically verified.
